# Prokofiev was (almost) right: A cross-cultural investigation of auditory-conceptual associations in *Peter and the Wolf*

**DOI:** 10.3758/s13423-023-02435-7

**Published:** 2024-01-24

**Authors:** Nicola Di Stefano, Alessandro Ansani, Andrea Schiavio, Charles Spence

**Affiliations:** 1 National Research Council, Institute of Cognitive Sciences and Technologies, Rome, Italy; 2 Centre of Excellence in Music, Mind, Body and Brain, Department of Music, Art and Culture Studies, Jyväskylä, Finland; 3 University of York, School of Arts and Creative Technologies, York, UK; 4 University of Oxford, Oxford, UK

**Keywords:** Music cognition, Sound recognition, Perceptual categorization and identification

## Abstract

Over recent decades, studies investigating cross-modal correspondences have documented the existence of a wide range of consistent cross-modal associations between simple auditory and visual stimuli or dimensions (e.g., pitch-lightness). Far fewer studies have investigated the association between complex and realistic auditory stimuli and visually presented concepts (e.g., musical excerpts-animals). Surprisingly, however, there is little evidence concerning the extent to which these associations are shared across cultures. To address this gap in the literature, two experiments using a set of stimuli based on Prokofiev’s symphonic fairy tale *Peter and the Wolf* are reported. In Experiment 1, 293 participants from several countries and with very different language backgrounds rated the association between the musical excerpts, images and words representing the story’s characters (namely, bird, duck, wolf, cat, and grandfather). The results revealed that participants tended to consistently associate the wolf and the bird with the corresponding musical excerpt, while the stimuli of other characters were not consistently matched across participants. Remarkably, neither the participants’ cultural background, nor their musical expertise affected the ratings. In Experiment 2, 104 participants were invited to rate each stimulus on eight emotional features. The results revealed that the emotional profiles associated with the music and with the concept of the wolf and the bird were perceived as more consistent between observers than the emotional profiles associated with the music and the concept of the duck, the cat, and the grandpa. Taken together, these findings therefore suggest that certain auditory-conceptual associations are perceived consistently across cultures and may be mediated by emotional associations.

**Supplementary Information:**

The online version contains supplementary material available at 10.3758/s13423-023-02435-7.

## Introduction

Many published studies have documented the existence of a variety of cross-modal correspondences between several sensory dimensions involving simple auditory and visual stimuli, such as pitch and size (e.g., Evans & Treisman, 2010; Gallace & Spence, 2006; Mondloch & Maurer, 2004), pitch and timbre/textural features of sound, such as roughness (e.g., Eitan & Timmers, 2010; Hamilton-Fletcher et al., 2018; see Di Stefano & Spence, 2022, for a review on roughness), lightness and brightness (e.g., Brunel et al., 2015; Hubbard, 1996; Klapetek et al., 2012; Marks, 1974, 1987; Wallmark et al., 2021, hue (e.g., Melara, 1989; Griscom & Palmer, 2012; Spence & Di Stefano, 2022, for a review), shape/angularity (Marks, 1987; Parise & Spence, 2012). When it comes to understanding the origins of such cross-modal correspondences, scholars have often evoked basic sensory mechanisms, such as associative learning, statistical co-occurrence, and occasionally also perceptual similarity (see Spence, 2011, 2020, for reviews). Importantly, although the phenomenon of cross-modal associations might evoke the notion of synaesthesia, the two are quite different. While the former are experienced consistently by nonsynaesthetes (and, to some extent, by synaesthetes as well), the latter are experienced by synesthetes only based on the idiosyncratic matching across the senses (see Deroy & Spence, 2013; Spence & Di Stefano, 2023).

In parallel, other researchers have explored audiovisual correspondences using more complex and realistic stimuli (e.g., classical music excerpts and paintings; see Albertazzi et al., 2015). In such cases, the complexity of the stimuli means that it is harder to explain the correspondences based on specific individual auditory/visual physical stimulus attributes/dimensions (e.g., frequency or hue; see Duthie, 2013; Duthie & Duthie, 2015), thus leading researchers to suggest the existence of extra-musical features mediating those associations. One of the most powerful accounts of such correspondences is the ‘emotional mediation hypothesis’, according to which the stimuli in different sensory domains are more likely to be matched if they share similar affective meanings (see Spence, 2020, for a review).

Several studies investigating music–colour correspondences have suggested that emotional mediation might be the underlying explanation (e.g., Barbiere et al., 2007; Cutietta & Haggerty, 1987; Isbilen & Krumhansl, 2016; Karwoski & Odbert, 1938; Odbert et al., 1942). For example, Barbiere and colleagues invited 27 students to choose a colour and an emotion (happy, sad, angry) that matched with different musical excerpts from Grieg, Mussorgsky, and Barber. The results revealed that the participants’ agreement on the choice of colour was higher for those musical selections that were associated with the same emotion than for those songs that were associated with different emotions. In a similar fashion, Palmer and his colleagues (2013) were able to demonstrate that people consistently associate different classical orchestral music excerpts with different colour patches (see also Isbilen & Krumhansl, 2016, for similar results using Preludes from Bach’s *Well-Tempered Clavier*).

However, to date, far less research has explored the association between concepts and complex pieces of music. One exception comes from a study by Trainor and Trehub (1992), in which 4- to 6-year-old children matched pictorial representations (cards) representing a wolf, a bird, a cat, and a duck to musical excerpts taken from Prokofiev’s *Peter and the Wolf* that were intended to represent those animals musically. The study explored children’s understanding of referential (or extra-musical) meaning in music (Miller, 2021). The results revealed that children matched appropriate animal pictures to musical excerpts at a level that was significantly better than chance. The wolf and bird were matched more readily than the cat and duck excerpts (see also Moore et al., 1999, on Saint Saëns’ *Carnival of Animals*). More recently, Albertazzi et al. (2015) demonstrated the existence of consistent audiovisual associations between highly complex stimuli (i.e., paintings) and music excerpts from classical repertoire for guitar or transcriptions (e.g., Villa-Lobos, Albeniz). These associations have been explained using the semantic differential technique based on perceptual and emotional features (e.g., bright and calm, respectively; see also Cowles, 1935; Iosifyan et al., 2022; Miller, 2021; Spence, 2020).

Despite the relatively early interest in cross-cultural approaches to cross-modal associations (e.g., Osgood, 1960; Rogers & Ross, 1968; see Wan et al., 2014, for a more recent study), it is surprising to observe the limited number of empirical investigations that have attempted to explore how audiovisual correspondences are perceived across cultures (Bremner et al., 2013; Chen et al., 2016; Eitan, 2017, for a review; O'Boyle et al., 1987; see also Palmer et al., 2013, though participants from the USA and New Mexico probably share a number of cultural traits). One should, however, also acknowledge the extensive cross-cultural literature on sound symbolism, starting from the early investigations of Sapir (e.g., 1929) to more recent studies (e.g., Ćwiek et al., 2022; D’Anselmo et al., 2019; see Svantesson, 2017, for a review). However, this literature is only tangentially related to the present investigation as it does not consider musical sounds, being directed essentially at unravelling the allegedly natural connection between the sound of words and their associations.

The investigations conducted thus far have primarily employed simple audiovisual stimuli. Surprisingly, no studies have explored the impact of culture on audiovisual associations involving complex stimuli (though see the mentioned study by Palmer et al., 2013, for a possible exception for the musical stimuli). In an attempt to fill this gap in the empirical literature, the present work has a twofold aim: First, to provide a cross-cultural and also multilingual investigation of auditory-conceptual associations; Second, to test whether the emotional mediation hypothesis could account for the associations, if these were found to be consistent across cultures. To achieve these objectives, two experimental protocols were designed.

In Experiment 1, participants rated the extent to which different images, music, and words matched. The images depicted five of the characters from *Peter and the Wolf* (i.e., the bird, duck, wolf, cat, and grandpa); the audio stimuli reproduced the music the composer created to represent each character; the words were chosen to relate to the name of the five characters. In Experiment 2, a different sample of participants was invited to create an emotional profile for each of the stimuli used in Experiment 1 (i.e., images, music, and words), by rating each stimulus for what Palmer et al. (2013) classified as “emotional features” (i.e., happy, sad, angry, calm, strong, weak, lively, and dreary).

## Experiment 1. Audio-conceptual associations: Music, images, and words

### Methods

#### Participants

Two hundred and ninety-three participants (65.5% females, mean age: 33.81 ± 14.75 years) were recruited by the authors through personal contact networks (e.g., email and social media). All partial completions (i.e., completion rate < 100%) were discarded throughout data collection and prior to data analysis. The participants were recruited from different continents: 117 (39.9%) from Europe, 71 (24.2%) from Asia, 53 (18.1%) from North America, 45 (15.4%) from Latin America, and 1 (0.3%) from Africa. Participants were grouped into four samples according to the language they chose to run the protocol, namely English (*n* = 76, 25.9%), Italian (*n* = 88, 30.0%), Spanish (*n* = 55, 18.8%), and Chinese (*n* = 74, 25.3%). The study was approved by the Research Ethics and Integrity Committee of the National Research Council of Italy.

#### Stimuli

The audio stimuli consisted of five musical excerpts from Prokofiev’s *Peter and the Wolf* (Prokofiev, 1942) extracted from the musical presentation of the characters of the story that is included at the beginning of this symphonic fairy tale (Prokofiev, 1942, p. iv). The musical excerpts were associated with the following characters: bird, cat, duck, wolf and grandpa. Excerpts were saved as .wav files (stereo, 16-bit, 44.1 kHz). Visual stimuli consisted of five black and white stylized drawings representing each character (bird, cat, duck, wolf, and grandpa). While some of the chosen characters could presumably also have been represented by using other senses (e.g., touch, or perhaps smell), we decided to represent these characters visually because, perhaps influenced by the famous Walt Disney’s animated story released in 1946, a visual representation seems to be the most spontaneously associated to all these characters. For the semantic tasks, the chosen words were related to the original in different ways, namely, synonymic relationships (e.g., goose instead of duck), similarity (e.g., hyena instead of wolf), or relationships based on grammatical gender (e.g., grandmother instead of grandfather). (The visual stimuli are available in the Online Supplementary Material (OSM).)

#### Experimental procedure

The test was available in four languages – Italian, English, Spanish, and Chinese – and was administered through Qualtrics (qualtrics.com). The experimental procedure consisted of three tasks. The first one was the Image-Music association task (Task 1), in which the participants were presented with all possible image-music associations and were invited to rate the extent to which the musical excerpt matched with the image using a slider ranging from 0 (‘do not match at all’) to 100 (‘very good match’), described here as ‘Fit’. Once the participants had completed Task 1, they were then presented with two additional tasks in a random order. One was the Music-Image task (Task 2), in which they were presented with one music excerpt and the five images at the same time, and were invited to select which figure best matched the music. In the semantic task (Task 3), the participants were presented with a musical excerpt and five words and were invited to select a word that matched the musical excerpt.

### Results

All of the statistical analyses were run through IBM SPSS 27 (IBM, 2020) and Jamovi (The jamovi project, 2022). The models were implemented through the General Analyses for the Linear Model module in Jamovi (GAMLj; Gallucci, 2022). The significance level was set to α = .05. Due to the high number of pairwise comparisons, their significance levels have been adjusted using Bonferroni correction in order to control the occurrence of false positives (Abdi, 2007; Haynes, 2013). In the *Results* section, the means (Ms) and probabilities (Ps) are accompanied by their 95% confidence intervals (95% CIs).

#### Task 1: *Image-music association*

A significant Kolmogorov-Smirnov test (*p* < .001) verified that the Fit variable showed significant departure from a normal distribution (Skewness = .05; Kurtosis = −1.36). For this reason, the associations were measured by means of a Generalized Linear Mixed-effect model (GLMM; Stroup, 2013) with Gamma distribution and the inverse link function. Such a configuration has proven to be effective when dealing with a dependent variable that has a positive skew and a continuous, non-negative range (Dunn & Smyth, 2018; Ng & Cribbie, 2017). The dependent variable of the model was the Fit score assigned to each audiovisual association (ranging from 0 to 100). To control the wide variability generated by our participants’ different backgrounds and musical expertise, participants were modelled as random intercepts. This approach helps in considering and controlling for the individual differences among participants when analysing the data. Random intercepts allow the statistical model to accommodate and adjust for the inherent variability among participants, thus ensuring that the results are not overly influenced by these differences (for a detailed explanation of random effects, see Kain et al., 2015). All three of the fixed effects computed were statistically significant: namely, music (*χ*^*2*^ = 75, *df* = 4, *p* < .001), image (*χ*^*2*^ = 160.6, *df* = 4, *p* < .001), and their interaction (*χ*^*2*^ = 454.8, *df* = 16, *p* < .001). The image of the cat achieved the highest Fit score (*M* = 49.7, 95% CI [46.3, 53.7] *SE* = 1.88), whereas the image of the bird had the lowest score (*M* = 30.4, 95% CI [28.1, 33.1] *SE* = 1.27) (see OSM, Table 1, for complete scores and Bonferroni-corrected pairwise contrasts). Regarding the musical excerpts, the highest Fit scores were associated to the cat melody (*M* = 54.6, 95% CI [50.8, 59.0] *SE* = 2.08), whereas the lowest score was related to the wolf excerpt (*M* = 27.3, 95% CI [25.3, 29.6] *SE* = 1.10).

**Fig. 1:**
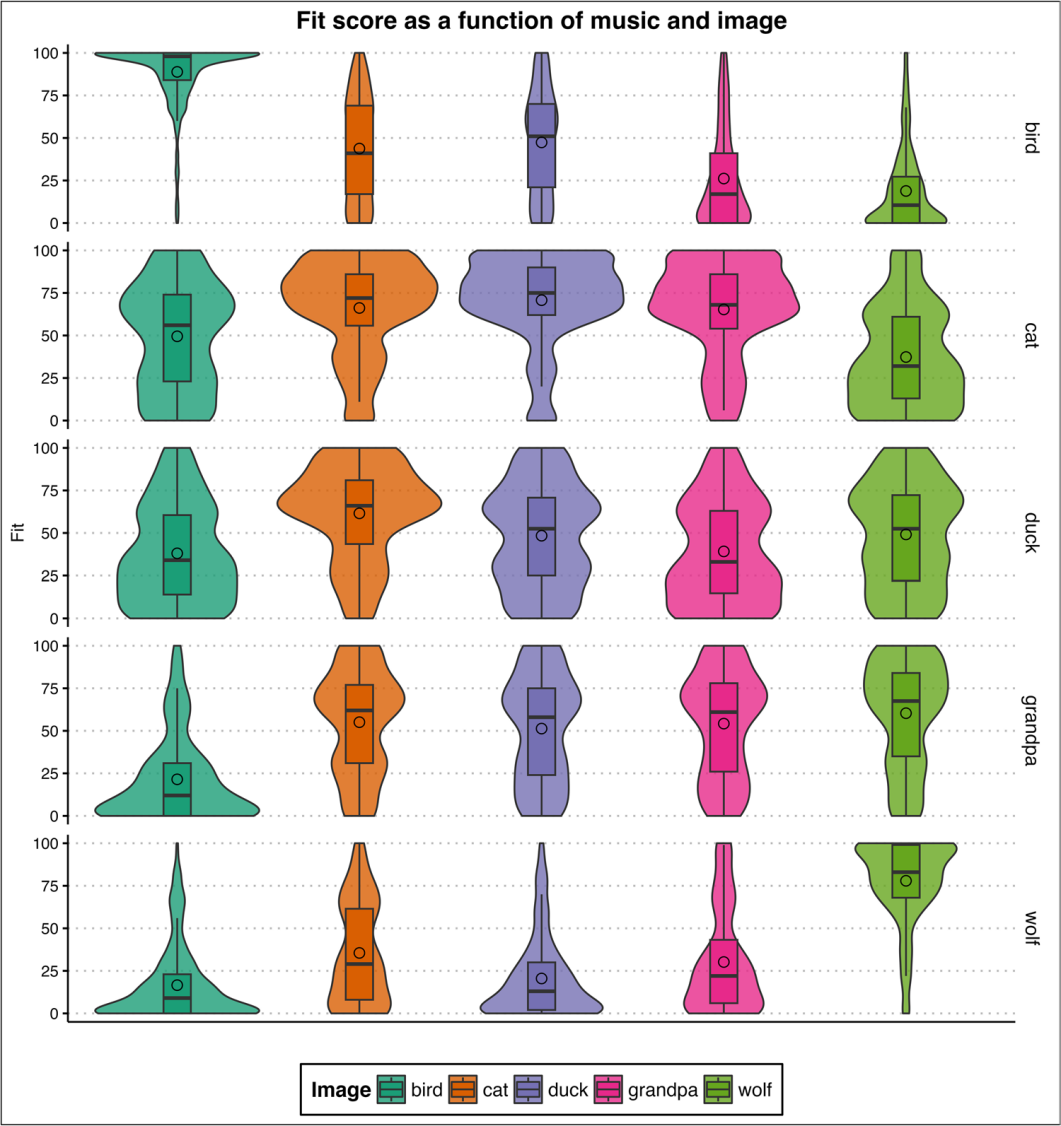
Fit score as a function of Image and Music. In the y-axis, for each Image-Music coupling, 100 means that participants rated the coupling as ‘very good’, while 0 indicates that the two stimuli ‘do not match’. The form of the violin plots indicates the distribution curve. The boxplots within each violin represent interquartile ranges (IQRs). Black horizontal lines within the boxplots indicate median values. Black circles within the boxplots indicate mean values. Horizontal layers are the different musical excerpts, colours represent images

The most interesting result is the interaction effect given the particular research question addressed here (see Fig. 1 for the results; see also OSM, Table 2).

A clear pattern emerged when comparing the Fit scores of the ‘correct’ couplings (i.e., between the image of the character and music according to the intention of the composer): the bird (*M* = 88.8, 95% CI [76.5, 105.9] *SE* = 7.30) and wolf (*M* = 77.9, 95% CI [67.1, 92.9] *SE* = 6.42) performed very well. The correct matching of cat image-melody also performed well (*M* = 66.2, 95% CI [57.0, 78.9] *SE* = 5.45). The cat melody is judged numerically as a better fit to the duck image (*M* = 70.7, 95% CI [60.9, 84.3] *SE* = 5.82), although this difference is not significant (*p* = .565), and the fit is not different than to the grandpa image (*M* = 65.3, 95% CI [56.3, 77.9] *SE* = 5.37, *p* = .912).

To explore the differences between those participants who had some knowledge of *Peter and the Wolf* and those who had none, the GLMM model was duplicated. One model was used for the former participants, the other for the latter. The results of the two models overlapped, suggesting that familiarity with music did not affect the way in which the participants associated music to images. Similar considerations hold for the comparison amongst different background languages/culture, with no evidence of any influence of cultural traits on participants’ choice (see OSM, Fig. 1).

#### Task 2: *Music-image association*

To investigate how participants associated images to musical excerpts, due to the multinomial nature of our dependent variable, a Generalized Multinomial Logit Model (G-MNL) was used (Fiebig et al., 2010). Compared to a regular Multinomial regression, this method has the advantage of not assuming the independence of irrelevant alternatives. This means that, in the context of G-MNL, the odds of choosing one category over another can be influenced by the presence of other categories. Furthermore, in contrast to Multinomial regression, G-MNLs allow for correlations between categories and are therefore more flexible in modelling complex choice behaviours. The chosen image was the dependent variable, whereas the music excerpts was the main fixed factor assessed. However, given that the order of presentation of Tasks 2 and 3 was randomized, we also assessed the effect of presentation order and the interaction Musical excerpt × Presentation order. A log-likelihood ratio test^1^ proved that the effect of the Musical excerpt was significant (*χ*^*2*^ = 1753.86, *df* = 16, *p* < .001), whereas the effect of Presentation order (*χ*^*2*^ = 6.38, *df* = 4, *p* = .173) and interaction (*χ*^*2*^ = 18.20, *df* = 16, *p* = .312) were not (see OSM, Table 3, for the probability assigned to all image-excerpts associations).

The results are consistent with those of Task 1; namely, the bird image was the most likely to be chosen when participants are asked to associate an image to the bird melody (*P* = 93.5%, 95% CI [90.5, 96.4] *SE* = 1.4) and the same happens with the wolf image (*P* = 86.4%, 95% CI [82.3, 90.4] *SE* = 2.0), which was by far the most likely to be associated with the wolf excerpt. Conversely, the musical excerpts of the cat and duck were systematically confounded with each other. When presented with the cat melody, the participants chose the duck image significantly more often (*P* = 41.8%, 95% CI [35.9, 47.6] *SE* = 2.8) as opposed to all other images (*p* < .001).^2^ The probability of choosing a cat image (*P* = 27.0%, 95% CI [21.7, 32.2] *SE* = 2.6) was not different from the grandpa image (*p* = 1). When presented with the duck melody, the cat image was chosen most often (*P* = 48.4%, 95% CI [42.5, 54.2] *SE* = 2.8), statistically different from all the other estimated probabilities (*p* < .001) (see Table 1).

**Table 1:**
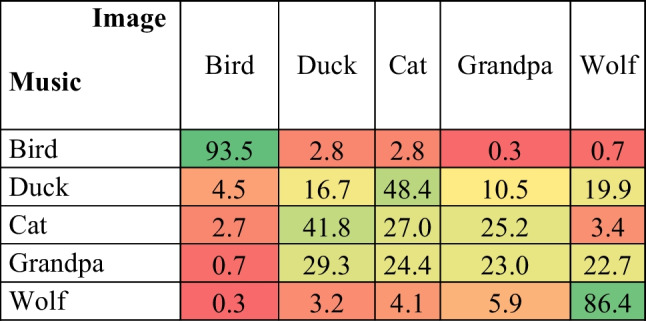
Confusion matrix representing the probability of choosing an image as a function of the musical excerpt. Colours represent the strength of the association, ranging from red (not associated) to green (most strongly associated)

#### Task 3: *Music-word association*

To investigate music-word association, a Generalized Multinomial Logit Model (G-MNL) was used (Fiebig et al., 2010) with the chosen word as the dependent variable. A log-likelihood ratio test proved that the effect of Music was significant (*χ*^*2*^ = 1674.15, *df* = 16, *p* < .001), as was the interaction effect (*χ*^*2*^ = 31.58, *df* = 16, *p* = .011), while there was no effect of Presentation order (*χ*^*2*^ = 5.95, *df* = 4, *p* = .203) (see OSM, Table 4, for details). The bird (*P* = 91.8%, 95% CI [88.5, 95.0] *SE* = 1.6) and wolf (*P* = 87.0%, 95% CI [83.0, 90.9] *SE* = 2.0) excerpts were significantly more strongly associated with the corresponding words than to all other words (*p* < .001). The cat melody was associated to the word duck (*P* = 50.4%, 95% CI [44.4, 56.3] *SE* = 2.9) and, vice versa, the duck melody was more likely to be related to the cat image (*P* = 39.7%, 95% CI [33.8, 45.4] *SE* = 2.8). Surprisingly, and differently from Task 2, the grandpa melody was associated most with the word ‘wolf’ (*P* = 34.8%, 95% CI [29.2, 40.4] *SE* = 2.7), and second to the word ‘grandpa’ (*P* = 30.4%, 95% CI [24.9, 35.8] *SE* = 2.7); the difference not being statistically significant (*p* = 1) (see Table 2).

**Table 2:**
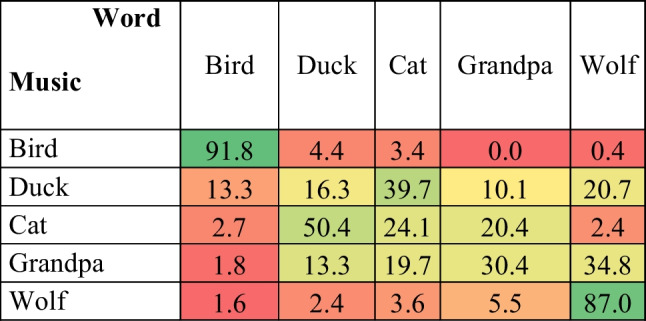
Confusion matrix representing the probability of choosing a word as a function of musical excerpt. The colours represent the strength of the association, ranging from red (not associated) to green (most strongly associated)

### Discussion

The results of Experiment 1 show that music, images, and words associated with the wolf and bird were consensually (i.e., according to Prokofiev’s intentions) matched across tasks and participants. These results confirm previous findings on children (Trainor & Trehub, 1992) and provide a more fine-grained analysis of the different cross-modal associations in an adult population involving individuals from different cultural backgrounds/languages. In particular, the results from the Image-Music task reveal that the image of the cat had the highest Fit score, suggesting that it showed no privileged association with any of the presented musical excerpts. In contrast, the image of the bird exhibits the lowest Fit score, confirming that it tends not to be chosen as a possible match with musical excerpts other than the bird. Furthermore, our findings provide clear evidence that participants’ associations between the experimental stimuli across different senses (i.e., audition, vision) are not contingent upon the order of stimulus presentation, participants’ culture/language, their musical background, or even their familiarity with *Peter and the Wolf*. Interestingly, some “wrong” associations are also rated consensually (e.g., duck-cat).

## Experiment 2. Emotional profiling of the auditory and visual stimuli

### Methods

#### Participants

Twenty-four participants were recruited by the authors through personal contact networks (e.g., email and social media). An additional 80 participants were recruited through Prolific.co. There was a total of 104 valid participants (48.1% female, mean age: 38.73 ± 12.67 years). All partial completions (i.e., completion rate < 100%) were discarded throughout data collection and prior to data analysis. Participants were either native English or Italian speakers from Europe (*n* = 89, 85.6%), North America (*n* = 7, 6.7%), Australia (*n* = 6, 5.8%), Latin America (*n* = 1, 1.0%), and Asia (*n* = 1, 1.0%). The study was approved by the Research Ethics and Integrity Committee of the National Research Council of Italy.

#### Stimuli

Stimuli were the same used in Experiment 1.

#### Experimental procedure

The test was administered through Qualtrics and was available in two languages: English and Italian. The participants were invited to assess the emotional profiles of all stimuli (music, images, and words) through the eight descriptors used in Palmer et al. (2013), namely, happy, sad, angry, calm, strong, weak, lively, and dreary. Many different emotional features could have been selected for similar tasks (see Menninghaus et al., 2019). However, given the controversial nature of so-called ‘aesthetic emotions’ – i.e., emotions associated with aesthetic stimuli (see Janowski & Chełkowska-Zacharewicz, 2019, Skov & Nadal, 2020) – we decided to use the same features that were used in the study by Palmer et al. (2013) to produce comparable outcomes. The participants indicated the extent to which each descriptor fit with the stimulus using a slider ranging from −100 to +100.

### Results

The analytic strategy was akin to that used by Palmer et al. (2013). Metric Multidimensional Scaling (MDS) was performed (Borg et al., 2013; Hout et al., 2013) through IBM SPSS’s (IBM, 2020) PROXSCAL tool (Busing et al., 1997). In our context, MDS implies that those stimuli that share a similar emotional profile are represented in proximal areas within the 2D space, while those stimuli that have dissimilar emotional profiles are represented in distal regions in the Cartesian plane. Manhattan distance was used as a dissimilarity measure due to its better performance with high dimensionality (Aggarwal et al., 2001). In line with the relevant literature (Groenen & Van De Velden, 2016; Solaro, 2011), the goodness of fit indices of the three MDS models (e.g., music, images and words) are reported in terms of the Dispersion Accounted For (i.e., DAF index; Little, 2013, p. 250), Stress-I (Kruskal, 1964), and S-Stress (Takane et al., 1977). Values below .10 are considered acceptable, while values below .05 are good. According to Dugard et al. (2010, p. 275), a good fit in MDS is represented by stress values < .15 and DAF values close to 1.

#### Correlation of descriptors

Table 3 reports results of the analyses of the correlations between each couple of opposite emotional descriptors, as in Palmer et al. (2013). The normality of all descriptors was checked via the Shapiro-Wilk test. Given that all descriptors showed significant deviations from the normal distribution (i.e., all *p* values < .001), we resorted to Spearman’s rank correlational analysis.

**Table 3:** Spearman’s rank correlation analysis on the descriptors

	Happy/sad	Weak/strong	Angry/calm	Lively/dreary
Musical excerpts	−.76	−.57	−.27	−.65
Images	−.70	−.62	−.45	−.36
Words	−.53	−.74	−.38	−.37
Overall	−.69	−.64	−.39	−.46

The values represent Spearman’s ρ. All *p* values are < .001

#### Multidimensional scaling

**Fig. 2:**
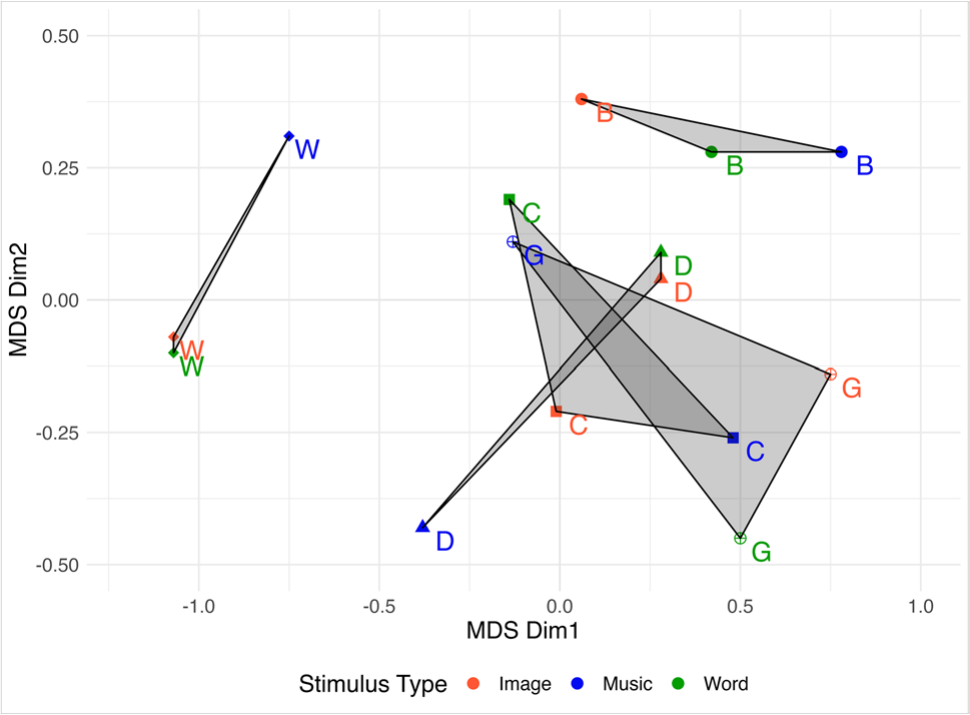
Two-dimensional (2D) map of the characters from the MDS distance matrix. The colours represent the stimulus type (i.e., images, music, and words). The different letters (B, C, D, G, W) represent the different stimuli (i.e., bird, cat, duck, grandpa, and wolf, respectively). Polygons represent the regions of the 2D space associated with each character.

Figure 2 shows the 2D solution obtained for music, images, and words accounted for 99% of the dispersion (i.e., DAF = .99, Stress-I = .027, S-stress = .001; DAF = .99, Stress-I = .028, S-stress = .002; DAF = .99, Stress-I = .021, S-stress = .001, for music, images, and words, respectively).

### Discussion

The results of Experiment 2 reveal that a clear emotional profile is associated with the wolf and the bird (see OSM, Fig. 2 for details on the emotional profile of each stimulus). As MDS shows (see Fig. 2), the polygons associated with the wolf and bird are distant from all of the other items as well as from each other. At the same time, the average Euclidean distance between the Image-Music and Word-Music of ‘wolf’ (0.509) and ‘bird’ (0.544) are lower than those associated with cat (0.630), duck (0.825) and grandpa (0.879). Moreover, the areas of duck, cat, and grandpa overlap one another, which means that, while the wolf and bird have a clear and mutually exclusive emotional profile, grandpa, duck, and cat share several emotional attributes thus making it hard for participants to clearly distinguish one from the other based on their affective meanings. This result might be related to the notion of “stimulus intricacy” which suggests that more intricate stimuli receive less consistent ratings across subjects according to different descriptors (Snitz et al., 2016).

## General discussion

Returning now to the study questions: First, were the associations put forward by Prokofiev perceived consistently across cultures/languages? As far as the wolf and bird characters are concerned, our findings suggest an affirmative response. However, the associations between music, images, and words for the duck, cat, and grandpa were not consistently perceived across participants. Second, were the highlighted associations mediated by emotional factors? Based on the results of Experiment 2, we might tentatively answer this question in the affirmative, as the emotional profile of the wolf and bird are quite clear with respect to those of cat, duck, and grandpa. This suggests that the characters of the wolf and bird and the music created to represent them have a similar emotional profile, and this might be the mediator for the cross-modal matchings that we reported in Experiment 1. In contrast, the characters of cat, duck, and grandpa and the music created to represent them have a less clear affective profile, which prevents them from being clearly associated on the basis of shared emotional features. One might then ask why bird and wolf are so special. It can be suggested that, in the experimental stimuli used here, wolf and bird represent contrasting features, both in terms of music (consonance vs. dissonance, major vs. minor, bright vs. dark timbre, low vs. high register, slow vs. fast tempo) and affective qualities (joyful vs. aggressive, day vs. night, light vs. dark).

The present findings lend support to the idea that complex audiovisual stimuli are more likely to elicit emotional meanings compared to simpler stimuli, such as isolated sounds. Therefore, the affective perspective previously outlined appears highly relevant in terms of understanding the associations between these stimuli (Spence, 2020; see also Cohen, 1993). That being said, we could not exclude the possibility that other factors might have played a role in mediating the associations, such as movement (jerky for the bird, slow for the wolf) or timbre (brilliant for the bird, dark for the wolf).

A more general caveat should also be made here, which regards the possibility that participants associated the stimuli based on the range of options that were available rather than a direct automatic cross-modal mapping (e.g., Schloss et al. 2018). The recent findings from Margulis et al. (2022) showing that free narratives imagined while listening to instrumental music are affected by cultural background might also suggest that the constrained nature of the matching tasks in our study played a role in determining the cross-cultural effect of certain cross-modal associations. Therefore, future works might replicate the same protocol to test whether audiovisual associations based on different musical compositions (e.g., Saint Saens’ *Carnival of Animals*), are consistently perceived across subjects and if they can be similarly mediated by affective qualities of the stimuli. Moreover, future investigations might extend to other cultures/languages, being thus able to eventually shed light on the related question on the existence of cultural variations in the way animal metaphors are conceived (e.g., see Sevillano & Fiske, 2019; Talebinejad & Dastjerdi, 2005).

### Supplementary information

ESM 1 (DOCX 2258 kb)

## Data Availability

The data that support the findings of this study are available from the corresponding author, N.D.S., upon reasonable request. None of the experiments was preregistered.

## Footnotes

1 In the context of a G-MNL, the log-likelihood ratio test verifies that at least one of the predictors’ coefficients is different from zero.

2 This *p* value refers to the Bonferroni-corrected pairwise post-hoc comparisons. The estimated probability of the duck image within the cat melody was significantly higher than those of all the other images. Namely, the *p* values of all contrasts were < .001.
