# Supplementary material for: Prokofiev was (almost) right: A cross-cultural investigation of auditory-conceptual associations in Peter and the Wolf
Source: Psychon Bull Rev. 2024 Jan 24;31(4):1735–44. doi: 10.3758/s13423-023-02435-7 (PMC11358347; doi:10.3758/s13423-023-02435-7)
Supplement: Supplementary file 1 — (DOCX 2258 kb) [file 13423_2023_2435_MOESM1_ESM.docx]

**Appendix to**

Prokofiev was (almost) right: A cross-cultural investigation

of auditory-conceptual associations in *Peter and the Wolf*

Nicola Di Stefano (corresponding author)

Institute of Cognitive Sciences and Technologies

National Research Council of Italy (CNR)

Via S. Martino della Battaglia 44, 00185 Roma (RM)

[nicola.distefano@istc.cnr.it](mailto:nicola.distefano@istc.cnr.it)

Alessandro Ansani (equal contribution)

Centre of Excellence in Music, Mind, Body and Brain

University of Jyväskylä, FI

Andrea Schiavio

School of Arts and Creative Technologies

University of York, UK

&

Charles Spence

Crossmodal Research Laboratory

University of Oxford, UK

**STIMULI**

The musical excerpts appear in the first page of the score (Prokovieff, 1942) after the following text: “Each character in this tale is represented by a different instrument of the orchestra: the Bird by the Flute, the Duck by the Oboe, the cat by the Clarinet, the Grandfather by the bassoon, the Wolf by the three Horns […] Before a performance with the orchestra it is desirable to show these instruments to the children and to play them the corresponding motives” (Prokofiev, 1942, p. iv). Although not considered a synaesthete, Prokofiev was one of the first and most prolific composers of music for visual art forms (from cinema to theatre). Interesting insights into Prokofiev’s approach to audiovisuals are provided by one of the film directors he collaborated with, namely Sergei Eisenstein, in his book *Nonindifferent Nature* (Eisenstein, 1945).

Eisenstein, S. (1988). Nonindifferent nature: Film and the structure of things. Cambridge, UK: Cambridge University Press.

Prokofiev, S. (1942). Peter and the Wolf. A Musical Tale for Children. Op. 67. London, UK: Hawkes & Son.

1. **Visual stimuli**


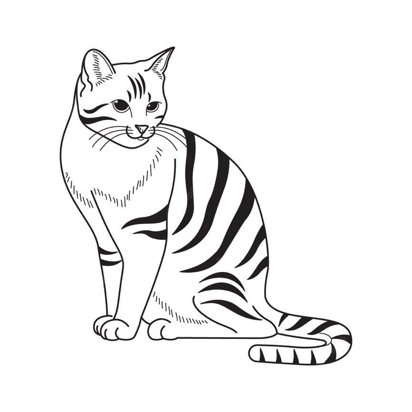

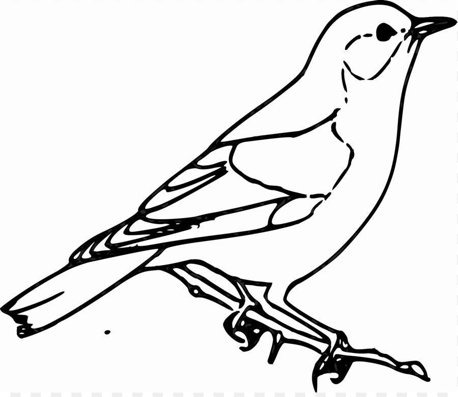

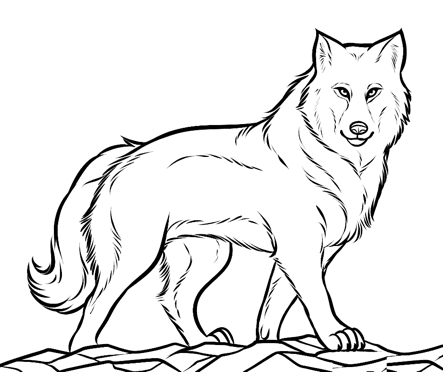


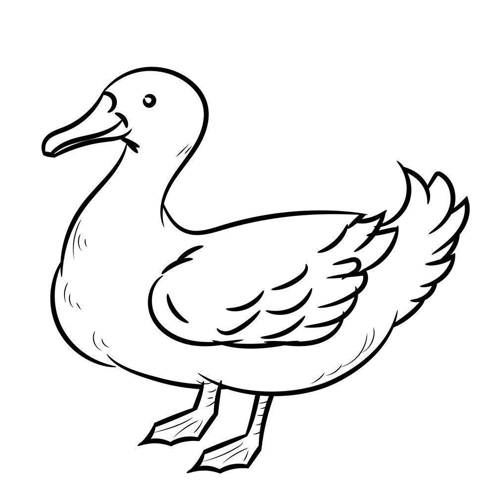

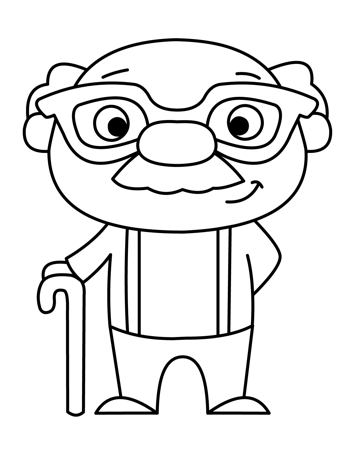


1. **Words**

| **English** | **Italian** | **Spanish** | **Chinese** |
| --- | --- | --- | --- |
| Sparrow, goldfinch, nightingale, finch, robin | Cardellino, passerotto, usignolo, fringuello, pettirosso | Jilguero, [gorrión, ruiseñor, pinzón, petirrojo](https://context.reverso.net/traduzione/spagnolo-italiano/gorri%C3%B3n) | 麻雀, 金翅雀, 夜莺, 雀科, 知更鸟 |
| Cat, kitten, feline, kitty, pussycat | Gatto, micio, felino, gatta, gattino | Gato, gatito, feline, gata, minino | 猫, 小猫, 猫科动物, 猫咪, 女猫 |
| Duck, duckling, goose, chick, gosling | Anatra, papera, oca, anatroccolo, papero | Pato, ganso, oca, patito, ansarino | 鸭, 小鸭子, 鹅, 小鸡, 小鹅 |
| Grandpa, granny, grandfather, old man, grandmother | Nonno, vecchietto, uomo anziano, nonna, donna anziana | Abuelo, viejitos, hombre anciano, abuela, mujer anciana | 奶奶, 爷爷, 祖父, 老人, 祖母 |
| Wolf, predator, hunting animal, panther, hyena | Lupo, animale predatore, animale cacciatore, pantera, iena | Lobo, animal depredador, animal cazador, pantera, hiena | 狼, 捕食动物, 狩猎动物, 豹, 鬣狗 |

**RESULTS**

**Table 1.** Post hoc comparisons Image x Melody. Bonferroni-corrected pairwise comparisons are reported in the p^‡^ column.

|  | | | | | | | | | | | | | | | | | | | |
| --- | --- | --- | --- | --- | --- | --- | --- | --- | --- | --- | --- | --- | --- | --- | --- | --- | --- | --- | --- |
| **Comparison** | | | | | | | | | |  | | | | | | | | | |
| **Image** | | **Melody** | |  | | **Image** | | **Melody** | | **Difference** | | **SE** | | **z** | | **p** | | **p^‡^** | |
| bird |  | bird |  | - |  | bird |  | cat |  | -0.009 |  | 0.002 |  | -4.692 |  | < .001 |  | < .001 |  |
| bird |  | bird |  | - |  | bird |  | duck |  | -0.015 |  | 0.002 |  | -6.379 |  | < .001 |  | < .001 |  |
| bird |  | bird |  | - |  | bird |  | grandpa |  | -0.035 |  | 0.004 |  | -8.962 |  | < .001 |  | < .001 |  |
| bird |  | bird |  | - |  | bird |  | wolf |  | -0.049 |  | 0.005 |  | -9.723 |  | < .001 |  | < .001 |  |
| bird |  | bird |  | - |  | cat |  | bird |  | -0.012 |  | 0.002 |  | -5.535 |  | < .001 |  | < .001 |  |
| bird |  | bird |  | - |  | cat |  | cat |  | -0.004 |  | 0.002 |  | -2.488 |  | 0.013 |  | 1.000 |  |
| bird |  | bird |  | - |  | cat |  | duck |  | -0.005 |  | 0.002 |  | -3.065 |  | 0.002 |  | 0.653 |  |
| bird |  | bird |  | - |  | cat |  | grandpa |  | -0.007 |  | 0.002 |  | -3.930 |  | < .001 |  | 0.025 |  |
| bird |  | bird |  | - |  | cat |  | wolf |  | -0.017 |  | 0.002 |  | -6.758 |  | < .001 |  | < .001 |  |
| bird |  | bird |  | - |  | duck |  | bird |  | -0.010 |  | 0.002 |  | -5.008 |  | < .001 |  | < .001 |  |
| bird |  | bird |  | - |  | duck |  | cat |  | -0.003 |  | 0.001 |  | -1.938 |  | 0.053 |  | 1.000 |  |
| bird |  | bird |  | - |  | duck |  | duck |  | -0.009 |  | 0.002 |  | -4.845 |  | < .001 |  | < .001 |  |
| bird |  | bird |  | - |  | duck |  | grandpa |  | -0.008 |  | 0.002 |  | -4.439 |  | < .001 |  | 0.003 |  |
| bird |  | bird |  | - |  | duck |  | wolf |  | -0.037 |  | 0.004 |  | -9.117 |  | < .001 |  | < .001 |  |
| bird |  | bird |  | - |  | grandpa |  | bird |  | -0.027 |  | 0.003 |  | -8.235 |  | < .001 |  | < .001 |  |
| bird |  | bird |  | - |  | grandpa |  | cat |  | -0.004 |  | 0.002 |  | -2.596 |  | 0.009 |  | 1.000 |  |
| bird |  | bird |  | - |  | grandpa |  | duck |  | -0.014 |  | 0.002 |  | -6.217 |  | < .001 |  | < .001 |  |
| bird |  | bird |  | - |  | grandpa |  | grandpa |  | -0.007 |  | 0.002 |  | -4.044 |  | < .001 |  | 0.016 |  |
| bird |  | bird |  | - |  | grandpa |  | wolf |  | -0.022 |  | 0.003 |  | -7.592 |  | < .001 |  | < .001 |  |
| bird |  | bird |  | - |  | wolf |  | bird |  | -0.042 |  | 0.004 |  | -9.358 |  | < .001 |  | < .001 |  |
| bird |  | bird |  | - |  | wolf |  | cat |  | -0.016 |  | 0.002 |  | -6.503 |  | < .001 |  | < .001 |  |
| bird |  | bird |  | - |  | wolf |  | duck |  | -0.009 |  | 0.002 |  | -4.751 |  | < .001 |  | < .001 |  |
| bird |  | bird |  | - |  | wolf |  | grandpa |  | -0.005 |  | 0.002 |  | -3.222 |  | 0.001 |  | 0.382 |  |
| bird |  | bird |  | - |  | wolf |  | wolf |  | -0.002 |  | 0.001 |  | -1.122 |  | 0.262 |  | 1.000 |  |
| bird |  | cat |  | - |  | bird |  | duck |  | -0.006 |  | 0.003 |  | -2.231 |  | 0.026 |  | 1.000 |  |
| bird |  | cat |  | - |  | bird |  | grandpa |  | -0.026 |  | 0.004 |  | -6.309 |  | < .001 |  | < .001 |  |
| bird |  | cat |  | - |  | bird |  | wolf |  | -0.040 |  | 0.005 |  | -7.665 |  | < .001 |  | < .001 |  |
| bird |  | cat |  | - |  | cat |  | cat |  | 0.005 |  | 0.002 |  | 2.445 |  | 0.014 |  | 1.000 |  |
| bird |  | cat |  | - |  | cat |  | duck |  | 0.004 |  | 0.002 |  | 1.848 |  | 0.065 |  | 1.000 |  |
| bird |  | cat |  | - |  | cat |  | grandpa |  | 0.002 |  | 0.002 |  | 0.913 |  | 0.361 |  | 1.000 |  |
| bird |  | cat |  | - |  | cat |  | wolf |  | -0.008 |  | 0.003 |  | -2.781 |  | 0.005 |  | 1.000 |  |
| bird |  | cat |  | - |  | duck |  | cat |  | 0.006 |  | 0.002 |  | 2.985 |  | 0.003 |  | 0.850 |  |
| bird |  | cat |  | - |  | duck |  | duck |  | -0.000 |  | 0.002 |  | -0.195 |  | 0.845 |  | 1.000 |  |
| bird |  | cat |  | - |  | duck |  | grandpa |  | 0.001 |  | 0.002 |  | 0.307 |  | 0.759 |  | 1.000 |  |
| bird |  | cat |  | - |  | duck |  | wolf |  | -0.029 |  | 0.004 |  | -6.579 |  | < .001 |  | < .001 |  |
| bird |  | cat |  | - |  | grandpa |  | cat |  | 0.005 |  | 0.002 |  | 2.341 |  | 0.019 |  | 1.000 |  |
| bird |  | cat |  | - |  | grandpa |  | duck |  | -0.005 |  | 0.003 |  | -1.990 |  | 0.047 |  | 1.000 |  |
| bird |  | cat |  | - |  | grandpa |  | grandpa |  | 0.002 |  | 0.002 |  | 0.782 |  | 0.434 |  | 1.000 |  |
| bird |  | cat |  | - |  | grandpa |  | wolf |  | -0.013 |  | 0.003 |  | -4.043 |  | < .001 |  | 0.016 |  |
| bird |  | cat |  | - |  | wolf |  | cat |  | -0.007 |  | 0.003 |  | -2.386 |  | 0.017 |  | 1.000 |  |
| bird |  | cat |  | - |  | wolf |  | duck |  | -0.000 |  | 0.002 |  | -0.064 |  | 0.949 |  | 1.000 |  |
| bird |  | cat |  | - |  | wolf |  | grandpa |  | 0.004 |  | 0.002 |  | 1.687 |  | 0.092 |  | 1.000 |  |
| bird |  | cat |  | - |  | wolf |  | wolf |  | 0.007 |  | 0.002 |  | 3.734 |  | < .001 |  | 0.056 |  |
| bird |  | duck |  | - |  | bird |  | grandpa |  | -0.020 |  | 0.004 |  | -4.598 |  | < .001 |  | 0.001 |  |
| bird |  | duck |  | - |  | bird |  | wolf |  | -0.034 |  | 0.005 |  | -6.280 |  | < .001 |  | < .001 |  |
| bird |  | duck |  | - |  | cat |  | duck |  | 0.010 |  | 0.003 |  | 3.943 |  | < .001 |  | 0.024 |  |
| bird |  | duck |  | - |  | cat |  | grandpa |  | 0.008 |  | 0.003 |  | 3.093 |  | 0.002 |  | 0.595 |  |
| bird |  | duck |  | - |  | cat |  | wolf |  | -0.002 |  | 0.003 |  | -0.580 |  | 0.562 |  | 1.000 |  |
| bird |  | duck |  | - |  | duck |  | duck |  | 0.006 |  | 0.003 |  | 2.042 |  | 0.041 |  | 1.000 |  |
| bird |  | duck |  | - |  | duck |  | grandpa |  | 0.007 |  | 0.003 |  | 2.523 |  | 0.012 |  | 1.000 |  |
| bird |  | duck |  | - |  | duck |  | wolf |  | -0.022 |  | 0.005 |  | -4.927 |  | < .001 |  | < .001 |  |
| bird |  | duck |  | - |  | grandpa |  | duck |  | 0.001 |  | 0.003 |  | 0.253 |  | 0.800 |  | 1.000 |  |
| bird |  | duck |  | - |  | grandpa |  | grandpa |  | 0.008 |  | 0.003 |  | 2.972 |  | 0.003 |  | 0.888 |  |
| bird |  | duck |  | - |  | grandpa |  | wolf |  | -0.007 |  | 0.003 |  | -1.956 |  | 0.050 |  | 1.000 |  |
| bird |  | duck |  | - |  | wolf |  | duck |  | 0.006 |  | 0.003 |  | 2.171 |  | 0.030 |  | 1.000 |  |
| bird |  | duck |  | - |  | wolf |  | grandpa |  | 0.010 |  | 0.003 |  | 3.799 |  | < .001 |  | 0.044 |  |
| bird |  | duck |  | - |  | wolf |  | wolf |  | 0.013 |  | 0.002 |  | 5.582 |  | < .001 |  | < .001 |  |
| bird |  | grandpa |  | - |  | bird |  | wolf |  | -0.014 |  | 0.006 |  | -2.198 |  | 0.028 |  | 1.000 |  |
| bird |  | grandpa |  | - |  | cat |  | grandpa |  | 0.028 |  | 0.004 |  | 6.908 |  | < .001 |  | < .001 |  |
| bird |  | grandpa |  | - |  | cat |  | wolf |  | 0.018 |  | 0.004 |  | 4.106 |  | < .001 |  | 0.012 |  |
| bird |  | grandpa |  | - |  | duck |  | grandpa |  | 0.027 |  | 0.004 |  | 6.515 |  | < .001 |  | < .001 |  |
| bird |  | grandpa |  | - |  | duck |  | wolf |  | -0.002 |  | 0.006 |  | -0.404 |  | 0.686 |  | 1.000 |  |
| bird |  | grandpa |  | - |  | grandpa |  | grandpa |  | 0.028 |  | 0.004 |  | 6.826 |  | < .001 |  | < .001 |  |
| bird |  | grandpa |  | - |  | grandpa |  | wolf |  | 0.013 |  | 0.005 |  | 2.852 |  | 0.004 |  | 1.000 |  |
| bird |  | grandpa |  | - |  | wolf |  | grandpa |  | 0.030 |  | 0.004 |  | 7.376 |  | < .001 |  | < .001 |  |
| bird |  | grandpa |  | - |  | wolf |  | wolf |  | 0.034 |  | 0.004 |  | 8.490 |  | < .001 |  | < .001 |  |
| bird |  | wolf |  | - |  | cat |  | wolf |  | 0.032 |  | 0.005 |  | 5.871 |  | < .001 |  | < .001 |  |
| bird |  | wolf |  | - |  | duck |  | wolf |  | 0.012 |  | 0.006 |  | 1.808 |  | 0.071 |  | 1.000 |  |
| bird |  | wolf |  | - |  | grandpa |  | wolf |  | 0.027 |  | 0.006 |  | 4.801 |  | < .001 |  | < .001 |  |
| bird |  | wolf |  | - |  | wolf |  | wolf |  | 0.047 |  | 0.005 |  | 9.363 |  | < .001 |  | < .001 |  |
| cat |  | bird |  | - |  | bird |  | cat |  | 0.003 |  | 0.003 |  | 1.053 |  | 0.292 |  | 1.000 |  |
| cat |  | bird |  | - |  | bird |  | duck |  | -0.003 |  | 0.003 |  | -1.206 |  | 0.228 |  | 1.000 |  |
| cat |  | bird |  | - |  | bird |  | grandpa |  | -0.024 |  | 0.004 |  | -5.557 |  | < .001 |  | < .001 |  |
| cat |  | bird |  | - |  | bird |  | wolf |  | -0.037 |  | 0.005 |  | -7.063 |  | < .001 |  | < .001 |  |
| cat |  | bird |  | - |  | cat |  | cat |  | 0.008 |  | 0.002 |  | 3.430 |  | < .001 |  | 0.181 |  |
| cat |  | bird |  | - |  | cat |  | duck |  | 0.007 |  | 0.002 |  | 2.859 |  | 0.004 |  | 1.000 |  |
| cat |  | bird |  | - |  | cat |  | grandpa |  | 0.005 |  | 0.002 |  | 1.953 |  | 0.051 |  | 1.000 |  |
| cat |  | bird |  | - |  | cat |  | wolf |  | -0.005 |  | 0.003 |  | -1.776 |  | 0.076 |  | 1.000 |  |
| cat |  | bird |  | - |  | duck |  | bird |  | 0.002 |  | 0.003 |  | 0.682 |  | 0.496 |  | 1.000 |  |
| cat |  | bird |  | - |  | duck |  | cat |  | 0.009 |  | 0.002 |  | 3.944 |  | < .001 |  | 0.024 |  |
| cat |  | bird |  | - |  | duck |  | duck |  | 0.002 |  | 0.003 |  | 0.858 |  | 0.391 |  | 1.000 |  |
| cat |  | bird |  | - |  | duck |  | grandpa |  | 0.003 |  | 0.002 |  | 1.357 |  | 0.175 |  | 1.000 |  |
| cat |  | bird |  | - |  | duck |  | wolf |  | -0.026 |  | 0.004 |  | -5.855 |  | < .001 |  | < .001 |  |
| cat |  | bird |  | - |  | grandpa |  | bird |  | -0.015 |  | 0.004 |  | -4.206 |  | < .001 |  | 0.008 |  |
| cat |  | bird |  | - |  | grandpa |  | cat |  | 0.008 |  | 0.002 |  | 3.332 |  | < .001 |  | 0.259 |  |
| cat |  | bird |  | - |  | grandpa |  | duck |  | -0.003 |  | 0.003 |  | -0.956 |  | 0.339 |  | 1.000 |  |
| cat |  | bird |  | - |  | grandpa |  | grandpa |  | 0.004 |  | 0.002 |  | 1.826 |  | 0.068 |  | 1.000 |  |
| cat |  | bird |  | - |  | grandpa |  | wolf |  | -0.010 |  | 0.003 |  | -3.104 |  | 0.002 |  | 0.574 |  |
| cat |  | bird |  | - |  | wolf |  | bird |  | -0.030 |  | 0.005 |  | -6.353 |  | < .001 |  | < .001 |  |
| cat |  | bird |  | - |  | wolf |  | cat |  | -0.004 |  | 0.003 |  | -1.363 |  | 0.173 |  | 1.000 |  |
| cat |  | bird |  | - |  | wolf |  | duck |  | 0.002 |  | 0.003 |  | 0.990 |  | 0.322 |  | 1.000 |  |
| cat |  | bird |  | - |  | wolf |  | grandpa |  | 0.006 |  | 0.002 |  | 2.704 |  | 0.007 |  | 1.000 |  |
| cat |  | bird |  | - |  | wolf |  | wolf |  | 0.010 |  | 0.002 |  | 4.647 |  | < .001 |  | 0.001 |  |
| cat |  | cat |  | - |  | bird |  | duck |  | -0.011 |  | 0.003 |  | -4.473 |  | < .001 |  | 0.002 |  |
| cat |  | cat |  | - |  | bird |  | grandpa |  | -0.031 |  | 0.004 |  | -7.808 |  | < .001 |  | < .001 |  |
| cat |  | cat |  | - |  | bird |  | wolf |  | -0.045 |  | 0.005 |  | -8.838 |  | < .001 |  | < .001 |  |
| cat |  | cat |  | - |  | cat |  | duck |  | -0.001 |  | 0.002 |  | -0.619 |  | 0.536 |  | 1.000 |  |
| cat |  | cat |  | - |  | cat |  | grandpa |  | -0.003 |  | 0.002 |  | -1.566 |  | 0.117 |  | 1.000 |  |
| cat |  | cat |  | - |  | cat |  | wolf |  | -0.013 |  | 0.003 |  | -4.947 |  | < .001 |  | < .001 |  |
| cat |  | cat |  | - |  | duck |  | cat |  | 0.001 |  | 0.002 |  | 0.576 |  | 0.565 |  | 1.000 |  |
| cat |  | cat |  | - |  | duck |  | duck |  | -0.006 |  | 0.002 |  | -2.627 |  | 0.009 |  | 1.000 |  |
| cat |  | cat |  | - |  | duck |  | grandpa |  | -0.004 |  | 0.002 |  | -2.151 |  | 0.031 |  | 1.000 |  |
| cat |  | cat |  | - |  | duck |  | wolf |  | -0.034 |  | 0.004 |  | -8.016 |  | < .001 |  | < .001 |  |
| cat |  | cat |  | - |  | grandpa |  | cat |  | -0.000 |  | 0.002 |  | -0.111 |  | 0.912 |  | 1.000 |  |
| cat |  | cat |  | - |  | grandpa |  | duck |  | -0.010 |  | 0.002 |  | -4.266 |  | < .001 |  | 0.006 |  |
| cat |  | cat |  | - |  | grandpa |  | grandpa |  | -0.003 |  | 0.002 |  | -1.694 |  | 0.090 |  | 1.000 |  |
| cat |  | cat |  | - |  | grandpa |  | wolf |  | -0.018 |  | 0.003 |  | -6.006 |  | < .001 |  | < .001 |  |
| cat |  | cat |  | - |  | wolf |  | cat |  | -0.012 |  | 0.003 |  | -4.615 |  | < .001 |  | 0.001 |  |
| cat |  | cat |  | - |  | wolf |  | duck |  | -0.005 |  | 0.002 |  | -2.508 |  | 0.012 |  | 1.000 |  |
| cat |  | cat |  | - |  | wolf |  | grandpa |  | -0.001 |  | 0.002 |  | -0.786 |  | 0.432 |  | 1.000 |  |
| cat |  | cat |  | - |  | wolf |  | wolf |  | 0.002 |  | 0.002 |  | 1.399 |  | 0.162 |  | 1.000 |  |
| cat |  | duck |  | - |  | bird |  | grandpa |  | -0.030 |  | 0.004 |  | -7.470 |  | < .001 |  | < .001 |  |
| cat |  | duck |  | - |  | bird |  | wolf |  | -0.044 |  | 0.005 |  | -8.576 |  | < .001 |  | < .001 |  |
| cat |  | duck |  | - |  | cat |  | grandpa |  | -0.002 |  | 0.002 |  | -0.952 |  | 0.341 |  | 1.000 |  |
| cat |  | duck |  | - |  | cat |  | wolf |  | -0.012 |  | 0.003 |  | -4.439 |  | < .001 |  | 0.003 |  |
| cat |  | duck |  | - |  | duck |  | duck |  | -0.004 |  | 0.002 |  | -2.035 |  | 0.042 |  | 1.000 |  |
| cat |  | duck |  | - |  | duck |  | grandpa |  | -0.003 |  | 0.002 |  | -1.549 |  | 0.121 |  | 1.000 |  |
| cat |  | duck |  | - |  | duck |  | wolf |  | -0.033 |  | 0.004 |  | -7.692 |  | < .001 |  | < .001 |  |
| cat |  | duck |  | - |  | grandpa |  | duck |  | -0.009 |  | 0.002 |  | -3.726 |  | < .001 |  | 0.058 |  |
| cat |  | duck |  | - |  | grandpa |  | grandpa |  | -0.002 |  | 0.002 |  | -1.083 |  | 0.279 |  | 1.000 |  |
| cat |  | duck |  | - |  | grandpa |  | wolf |  | -0.017 |  | 0.003 |  | -5.553 |  | < .001 |  | < .001 |  |
| cat |  | duck |  | - |  | wolf |  | duck |  | -0.004 |  | 0.002 |  | -1.912 |  | 0.056 |  | 1.000 |  |
| cat |  | duck |  | - |  | wolf |  | grandpa |  | -0.000 |  | 0.002 |  | -0.167 |  | 0.867 |  | 1.000 |  |
| cat |  | duck |  | - |  | wolf |  | wolf |  | 0.003 |  | 0.002 |  | 2.002 |  | 0.045 |  | 1.000 |  |
| cat |  | grandpa |  | - |  | bird |  | wolf |  | -0.042 |  | 0.005 |  | -8.138 |  | < .001 |  | < .001 |  |
| cat |  | grandpa |  | - |  | cat |  | wolf |  | -0.010 |  | 0.003 |  | -3.619 |  | < .001 |  | 0.089 |  |
| cat |  | grandpa |  | - |  | duck |  | grandpa |  | -0.001 |  | 0.002 |  | -0.607 |  | 0.544 |  | 1.000 |  |
| cat |  | grandpa |  | - |  | duck |  | wolf |  | -0.031 |  | 0.004 |  | -7.154 |  | < .001 |  | < .001 |  |
| cat |  | grandpa |  | - |  | grandpa |  | grandpa |  | -0.000 |  | 0.002 |  | -0.132 |  | 0.895 |  | 1.000 |  |
| cat |  | grandpa |  | - |  | grandpa |  | wolf |  | -0.015 |  | 0.003 |  | -4.813 |  | < .001 |  | < .001 |  |
| cat |  | grandpa |  | - |  | wolf |  | grandpa |  | 0.002 |  | 0.002 |  | 0.787 |  | 0.431 |  | 1.000 |  |
| cat |  | grandpa |  | - |  | wolf |  | wolf |  | 0.005 |  | 0.002 |  | 2.913 |  | 0.004 |  | 1.000 |  |
| cat |  | wolf |  | - |  | duck |  | wolf |  | -0.021 |  | 0.005 |  | -4.449 |  | < .001 |  | 0.003 |  |
| cat |  | wolf |  | - |  | grandpa |  | wolf |  | -0.005 |  | 0.004 |  | -1.388 |  | 0.165 |  | 1.000 |  |
| cat |  | wolf |  | - |  | wolf |  | wolf |  | 0.015 |  | 0.003 |  | 6.004 |  | < .001 |  | < .001 |  |
| duck |  | bird |  | - |  | bird |  | cat |  | 0.001 |  | 0.002 |  | 0.375 |  | 0.708 |  | 1.000 |  |
| duck |  | bird |  | - |  | bird |  | duck |  | -0.005 |  | 0.003 |  | -1.873 |  | 0.061 |  | 1.000 |  |
| duck |  | bird |  | - |  | bird |  | grandpa |  | -0.025 |  | 0.004 |  | -6.052 |  | < .001 |  | < .001 |  |
| duck |  | bird |  | - |  | bird |  | wolf |  | -0.039 |  | 0.005 |  | -7.461 |  | < .001 |  | < .001 |  |
| duck |  | bird |  | - |  | cat |  | cat |  | 0.006 |  | 0.002 |  | 2.804 |  | 0.005 |  | 1.000 |  |
| duck |  | bird |  | - |  | cat |  | duck |  | 0.005 |  | 0.002 |  | 2.214 |  | 0.027 |  | 1.000 |  |
| duck |  | bird |  | - |  | cat |  | grandpa |  | 0.003 |  | 0.002 |  | 1.286 |  | 0.198 |  | 1.000 |  |
| duck |  | bird |  | - |  | cat |  | wolf |  | -0.007 |  | 0.003 |  | -2.431 |  | 0.015 |  | 1.000 |  |
| duck |  | bird |  | - |  | duck |  | cat |  | 0.007 |  | 0.002 |  | 3.337 |  | < .001 |  | 0.254 |  |
| duck |  | bird |  | - |  | duck |  | duck |  | 0.000 |  | 0.002 |  | 0.179 |  | 0.858 |  | 1.000 |  |
| duck |  | bird |  | - |  | duck |  | grandpa |  | 0.002 |  | 0.002 |  | 0.682 |  | 0.495 |  | 1.000 |  |
| duck |  | bird |  | - |  | duck |  | wolf |  | -0.028 |  | 0.004 |  | -6.332 |  | < .001 |  | < .001 |  |
| duck |  | bird |  | - |  | grandpa |  | bird |  | -0.017 |  | 0.004 |  | -4.775 |  | < .001 |  | < .001 |  |
| duck |  | bird |  | - |  | grandpa |  | cat |  | 0.006 |  | 0.002 |  | 2.702 |  | 0.007 |  | 1.000 |  |
| duck |  | bird |  | - |  | grandpa |  | duck |  | -0.004 |  | 0.003 |  | -1.628 |  | 0.104 |  | 1.000 |  |
| duck |  | bird |  | - |  | grandpa |  | grandpa |  | 0.003 |  | 0.002 |  | 1.156 |  | 0.248 |  | 1.000 |  |
| duck |  | bird |  | - |  | grandpa |  | wolf |  | -0.012 |  | 0.003 |  | -3.718 |  | < .001 |  | 0.060 |  |
| duck |  | bird |  | - |  | wolf |  | bird |  | -0.032 |  | 0.005 |  | -6.798 |  | < .001 |  | < .001 |  |
| duck |  | bird |  | - |  | wolf |  | cat |  | -0.006 |  | 0.003 |  | -2.029 |  | 0.042 |  | 1.000 |  |
| duck |  | bird |  | - |  | wolf |  | duck |  | 0.001 |  | 0.002 |  | 0.311 |  | 0.756 |  | 1.000 |  |
| duck |  | bird |  | - |  | wolf |  | grandpa |  | 0.005 |  | 0.002 |  | 2.055 |  | 0.040 |  | 1.000 |  |
| duck |  | bird |  | - |  | wolf |  | wolf |  | 0.008 |  | 0.002 |  | 4.072 |  | < .001 |  | 0.014 |  |
| duck |  | cat |  | - |  | bird |  | duck |  | -0.012 |  | 0.002 |  | -4.944 |  | < .001 |  | < .001 |  |
| duck |  | cat |  | - |  | bird |  | grandpa |  | -0.032 |  | 0.004 |  | -8.102 |  | < .001 |  | < .001 |  |
| duck |  | cat |  | - |  | bird |  | wolf |  | -0.046 |  | 0.005 |  | -9.064 |  | < .001 |  | < .001 |  |
| duck |  | cat |  | - |  | cat |  | duck |  | -0.002 |  | 0.002 |  | -1.191 |  | 0.234 |  | 1.000 |  |
| duck |  | cat |  | - |  | cat |  | grandpa |  | -0.004 |  | 0.002 |  | -2.127 |  | 0.033 |  | 1.000 |  |
| duck |  | cat |  | - |  | cat |  | wolf |  | -0.014 |  | 0.003 |  | -5.397 |  | < .001 |  | < .001 |  |
| duck |  | cat |  | - |  | duck |  | duck |  | -0.007 |  | 0.002 |  | -3.162 |  | 0.002 |  | 0.471 |  |
| duck |  | cat |  | - |  | duck |  | grandpa |  | -0.005 |  | 0.002 |  | -2.699 |  | 0.007 |  | 1.000 |  |
| duck |  | cat |  | - |  | duck |  | wolf |  | -0.035 |  | 0.004 |  | -8.297 |  | < .001 |  | < .001 |  |
| duck |  | cat |  | - |  | grandpa |  | cat |  | -0.001 |  | 0.002 |  | -0.687 |  | 0.492 |  | 1.000 |  |
| duck |  | cat |  | - |  | grandpa |  | duck |  | -0.011 |  | 0.002 |  | -4.747 |  | < .001 |  | < .001 |  |
| duck |  | cat |  | - |  | grandpa |  | grandpa |  | -0.004 |  | 0.002 |  | -2.253 |  | 0.024 |  | 1.000 |  |
| duck |  | cat |  | - |  | grandpa |  | wolf |  | -0.019 |  | 0.003 |  | -6.404 |  | < .001 |  | < .001 |  |
| duck |  | cat |  | - |  | wolf |  | cat |  | -0.013 |  | 0.002 |  | -5.082 |  | < .001 |  | < .001 |  |
| duck |  | cat |  | - |  | wolf |  | duck |  | -0.006 |  | 0.002 |  | -3.048 |  | 0.002 |  | 0.691 |  |
| duck |  | cat |  | - |  | wolf |  | grandpa |  | -0.002 |  | 0.002 |  | -1.357 |  | 0.175 |  | 1.000 |  |
| duck |  | cat |  | - |  | wolf |  | wolf |  | 0.001 |  | 0.002 |  | 0.830 |  | 0.406 |  | 1.000 |  |
| duck |  | duck |  | - |  | bird |  | grandpa |  | -0.026 |  | 0.004 |  | -6.173 |  | < .001 |  | < .001 |  |
| duck |  | duck |  | - |  | bird |  | wolf |  | -0.040 |  | 0.005 |  | -7.557 |  | < .001 |  | < .001 |  |
| duck |  | duck |  | - |  | cat |  | grandpa |  | 0.003 |  | 0.002 |  | 1.106 |  | 0.269 |  | 1.000 |  |
| duck |  | duck |  | - |  | cat |  | wolf |  | -0.007 |  | 0.003 |  | -2.596 |  | 0.009 |  | 1.000 |  |
| duck |  | duck |  | - |  | duck |  | grandpa |  | 0.001 |  | 0.002 |  | 0.501 |  | 0.616 |  | 1.000 |  |
| duck |  | duck |  | - |  | duck |  | wolf |  | -0.028 |  | 0.004 |  | -6.448 |  | < .001 |  | < .001 |  |
| duck |  | duck |  | - |  | grandpa |  | duck |  | -0.005 |  | 0.003 |  | -1.799 |  | 0.072 |  | 1.000 |  |
| duck |  | duck |  | - |  | grandpa |  | grandpa |  | 0.002 |  | 0.002 |  | 0.976 |  | 0.329 |  | 1.000 |  |
| duck |  | duck |  | - |  | grandpa |  | wolf |  | -0.012 |  | 0.003 |  | -3.871 |  | < .001 |  | 0.033 |  |
| duck |  | duck |  | - |  | wolf |  | duck |  | 0.000 |  | 0.002 |  | 0.131 |  | 0.895 |  | 1.000 |  |
| duck |  | duck |  | - |  | wolf |  | grandpa |  | 0.004 |  | 0.002 |  | 1.875 |  | 0.061 |  | 1.000 |  |
| duck |  | duck |  | - |  | wolf |  | wolf |  | 0.008 |  | 0.002 |  | 3.901 |  | < .001 |  | 0.029 |  |
| duck |  | grandpa |  | - |  | bird |  | wolf |  | -0.041 |  | 0.005 |  | -7.828 |  | < .001 |  | < .001 |  |
| duck |  | grandpa |  | - |  | cat |  | wolf |  | -0.009 |  | 0.003 |  | -3.066 |  | 0.002 |  | 0.651 |  |
| duck |  | grandpa |  | - |  | duck |  | wolf |  | -0.029 |  | 0.004 |  | -6.777 |  | < .001 |  | < .001 |  |
| duck |  | grandpa |  | - |  | grandpa |  | grandpa |  | 0.001 |  | 0.002 |  | 0.476 |  | 0.634 |  | 1.000 |  |
| duck |  | grandpa |  | - |  | grandpa |  | wolf |  | -0.014 |  | 0.003 |  | -4.306 |  | < .001 |  | 0.005 |  |
| duck |  | grandpa |  | - |  | wolf |  | grandpa |  | 0.003 |  | 0.002 |  | 1.386 |  | 0.166 |  | 1.000 |  |
| duck |  | grandpa |  | - |  | wolf |  | wolf |  | 0.007 |  | 0.002 |  | 3.461 |  | < .001 |  | 0.162 |  |
| duck |  | wolf |  | - |  | grandpa |  | wolf |  | 0.016 |  | 0.005 |  | 3.225 |  | 0.001 |  | 0.377 |  |
| duck |  | wolf |  | - |  | wolf |  | wolf |  | 0.036 |  | 0.004 |  | 8.667 |  | < .001 |  | < .001 |  |
| grandpa |  | bird |  | - |  | bird |  | cat |  | 0.018 |  | 0.004 |  | 5.072 |  | < .001 |  | < .001 |  |
| grandpa |  | bird |  | - |  | bird |  | duck |  | 0.012 |  | 0.004 |  | 3.127 |  | 0.002 |  | 0.530 |  |
| grandpa |  | bird |  | - |  | bird |  | grandpa |  | -0.008 |  | 0.005 |  | -1.670 |  | 0.095 |  | 1.000 |  |
| grandpa |  | bird |  | - |  | bird |  | wolf |  | -0.022 |  | 0.006 |  | -3.753 |  | < .001 |  | 0.052 |  |
| grandpa |  | bird |  | - |  | cat |  | cat |  | 0.023 |  | 0.003 |  | 6.841 |  | < .001 |  | < .001 |  |
| grandpa |  | bird |  | - |  | cat |  | duck |  | 0.022 |  | 0.003 |  | 6.437 |  | < .001 |  | < .001 |  |
| grandpa |  | bird |  | - |  | cat |  | grandpa |  | 0.020 |  | 0.003 |  | 5.772 |  | < .001 |  | < .001 |  |
| grandpa |  | bird |  | - |  | cat |  | wolf |  | 0.010 |  | 0.004 |  | 2.584 |  | 0.010 |  | 1.000 |  |
| grandpa |  | bird |  | - |  | duck |  | cat |  | 0.024 |  | 0.003 |  | 7.193 |  | < .001 |  | < .001 |  |
| grandpa |  | bird |  | - |  | duck |  | duck |  | 0.018 |  | 0.004 |  | 4.914 |  | < .001 |  | < .001 |  |
| grandpa |  | bird |  | - |  | duck |  | grandpa |  | 0.019 |  | 0.004 |  | 5.312 |  | < .001 |  | < .001 |  |
| grandpa |  | bird |  | - |  | duck |  | wolf |  | -0.011 |  | 0.005 |  | -2.063 |  | 0.039 |  | 1.000 |  |
| grandpa |  | bird |  | - |  | grandpa |  | cat |  | 0.023 |  | 0.003 |  | 6.772 |  | < .001 |  | < .001 |  |
| grandpa |  | bird |  | - |  | grandpa |  | duck |  | 0.013 |  | 0.004 |  | 3.360 |  | < .001 |  | 0.234 |  |
| grandpa |  | bird |  | - |  | grandpa |  | grandpa |  | 0.020 |  | 0.003 |  | 5.675 |  | < .001 |  | < .001 |  |
| grandpa |  | bird |  | - |  | grandpa |  | wolf |  | 0.005 |  | 0.004 |  | 1.232 |  | 0.218 |  | 1.000 |  |
| grandpa |  | bird |  | - |  | wolf |  | bird |  | -0.015 |  | 0.005 |  | -2.752 |  | 0.006 |  | 1.000 |  |
| grandpa |  | bird |  | - |  | wolf |  | cat |  | 0.011 |  | 0.004 |  | 2.985 |  | 0.003 |  | 0.850 |  |
| grandpa |  | bird |  | - |  | wolf |  | duck |  | 0.018 |  | 0.004 |  | 5.023 |  | < .001 |  | < .001 |  |
| grandpa |  | bird |  | - |  | wolf |  | grandpa |  | 0.022 |  | 0.003 |  | 6.326 |  | < .001 |  | < .001 |  |
| grandpa |  | bird |  | - |  | wolf |  | wolf |  | 0.025 |  | 0.003 |  | 7.662 |  | < .001 |  | < .001 |  |
| grandpa |  | cat |  | - |  | bird |  | duck |  | -0.011 |  | 0.003 |  | -4.382 |  | < .001 |  | 0.004 |  |
| grandpa |  | cat |  | - |  | bird |  | grandpa |  | -0.031 |  | 0.004 |  | -7.751 |  | < .001 |  | < .001 |  |
| grandpa |  | cat |  | - |  | bird |  | wolf |  | -0.045 |  | 0.005 |  | -8.794 |  | < .001 |  | < .001 |  |
| grandpa |  | cat |  | - |  | cat |  | duck |  | -0.001 |  | 0.002 |  | -0.509 |  | 0.611 |  | 1.000 |  |
| grandpa |  | cat |  | - |  | cat |  | grandpa |  | -0.003 |  | 0.002 |  | -1.458 |  | 0.145 |  | 1.000 |  |
| grandpa |  | cat |  | - |  | cat |  | wolf |  | -0.013 |  | 0.003 |  | -4.861 |  | < .001 |  | < .001 |  |
| grandpa |  | cat |  | - |  | duck |  | duck |  | -0.005 |  | 0.002 |  | -2.524 |  | 0.012 |  | 1.000 |  |
| grandpa |  | cat |  | - |  | duck |  | grandpa |  | -0.004 |  | 0.002 |  | -2.046 |  | 0.041 |  | 1.000 |  |
| grandpa |  | cat |  | - |  | duck |  | wolf |  | -0.033 |  | 0.004 |  | -7.961 |  | < .001 |  | < .001 |  |
| grandpa |  | cat |  | - |  | grandpa |  | duck |  | -0.010 |  | 0.002 |  | -4.173 |  | < .001 |  | 0.009 |  |
| grandpa |  | cat |  | - |  | grandpa |  | grandpa |  | -0.003 |  | 0.002 |  | -1.587 |  | 0.113 |  | 1.000 |  |
| grandpa |  | cat |  | - |  | grandpa |  | wolf |  | -0.018 |  | 0.003 |  | -5.929 |  | < .001 |  | < .001 |  |
| grandpa |  | cat |  | - |  | wolf |  | cat |  | -0.011 |  | 0.003 |  | -4.525 |  | < .001 |  | 0.002 |  |
| grandpa |  | cat |  | - |  | wolf |  | duck |  | -0.005 |  | 0.002 |  | -2.405 |  | 0.016 |  | 1.000 |  |
| grandpa |  | cat |  | - |  | wolf |  | grandpa |  | -0.001 |  | 0.002 |  | -0.677 |  | 0.499 |  | 1.000 |  |
| grandpa |  | cat |  | - |  | wolf |  | wolf |  | 0.002 |  | 0.002 |  | 1.509 |  | 0.131 |  | 1.000 |  |
| grandpa |  | duck |  | - |  | bird |  | grandpa |  | -0.021 |  | 0.004 |  | -4.807 |  | < .001 |  | < .001 |  |
| grandpa |  | duck |  | - |  | bird |  | wolf |  | -0.035 |  | 0.005 |  | -6.452 |  | < .001 |  | < .001 |  |
| grandpa |  | duck |  | - |  | cat |  | grandpa |  | 0.007 |  | 0.003 |  | 2.862 |  | 0.004 |  | 1.000 |  |
| grandpa |  | duck |  | - |  | cat |  | wolf |  | -0.003 |  | 0.003 |  | -0.833 |  | 0.405 |  | 1.000 |  |
| grandpa |  | duck |  | - |  | duck |  | grandpa |  | 0.006 |  | 0.003 |  | 2.286 |  | 0.022 |  | 1.000 |  |
| grandpa |  | duck |  | - |  | duck |  | wolf |  | -0.023 |  | 0.005 |  | -5.129 |  | < .001 |  | < .001 |  |
| grandpa |  | duck |  | - |  | grandpa |  | grandpa |  | 0.007 |  | 0.003 |  | 2.740 |  | 0.006 |  | 1.000 |  |
| grandpa |  | duck |  | - |  | grandpa |  | wolf |  | -0.008 |  | 0.003 |  | -2.201 |  | 0.028 |  | 1.000 |  |
| grandpa |  | duck |  | - |  | wolf |  | duck |  | 0.005 |  | 0.003 |  | 1.930 |  | 0.054 |  | 1.000 |  |
| grandpa |  | duck |  | - |  | wolf |  | grandpa |  | 0.009 |  | 0.003 |  | 3.579 |  | < .001 |  | 0.103 |  |
| grandpa |  | duck |  | - |  | wolf |  | wolf |  | 0.013 |  | 0.002 |  | 5.400 |  | < .001 |  | < .001 |  |
| grandpa |  | grandpa |  | - |  | bird |  | wolf |  | -0.042 |  | 0.005 |  | -8.073 |  | < .001 |  | < .001 |  |
| grandpa |  | grandpa |  | - |  | cat |  | wolf |  | -0.010 |  | 0.003 |  | -3.502 |  | < .001 |  | 0.139 |  |
| grandpa |  | grandpa |  | - |  | duck |  | wolf |  | -0.030 |  | 0.004 |  | -7.076 |  | < .001 |  | < .001 |  |
| grandpa |  | grandpa |  | - |  | grandpa |  | wolf |  | -0.015 |  | 0.003 |  | -4.706 |  | < .001 |  | < .001 |  |
| grandpa |  | grandpa |  | - |  | wolf |  | grandpa |  | 0.002 |  | 0.002 |  | 0.917 |  | 0.359 |  | 1.000 |  |
| grandpa |  | grandpa |  | - |  | wolf |  | wolf |  | 0.006 |  | 0.002 |  | 3.034 |  | 0.002 |  | 0.723 |  |
| grandpa |  | wolf |  | - |  | wolf |  | wolf |  | 0.020 |  | 0.003 |  | 6.937 |  | < .001 |  | < .001 |  |
| wolf |  | bird |  | - |  | bird |  | cat |  | 0.033 |  | 0.005 |  | 7.026 |  | < .001 |  | < .001 |  |
| wolf |  | bird |  | - |  | bird |  | duck |  | 0.027 |  | 0.005 |  | 5.485 |  | < .001 |  | < .001 |  |
| wolf |  | bird |  | - |  | bird |  | grandpa |  | 0.007 |  | 0.006 |  | 1.127 |  | 0.260 |  | 1.000 |  |
| wolf |  | bird |  | - |  | bird |  | wolf |  | -0.007 |  | 0.007 |  | -1.092 |  | 0.275 |  | 1.000 |  |
| wolf |  | bird |  | - |  | cat |  | cat |  | 0.038 |  | 0.005 |  | 8.351 |  | < .001 |  | < .001 |  |
| wolf |  | bird |  | - |  | cat |  | duck |  | 0.037 |  | 0.005 |  | 8.054 |  | < .001 |  | < .001 |  |
| wolf |  | bird |  | - |  | cat |  | grandpa |  | 0.035 |  | 0.005 |  | 7.558 |  | < .001 |  | < .001 |  |
| wolf |  | bird |  | - |  | cat |  | wolf |  | 0.025 |  | 0.005 |  | 5.035 |  | < .001 |  | < .001 |  |
| wolf |  | bird |  | - |  | duck |  | cat |  | 0.039 |  | 0.005 |  | 8.608 |  | < .001 |  | < .001 |  |
| wolf |  | bird |  | - |  | duck |  | duck |  | 0.032 |  | 0.005 |  | 6.905 |  | < .001 |  | < .001 |  |
| wolf |  | bird |  | - |  | duck |  | grandpa |  | 0.034 |  | 0.005 |  | 7.210 |  | < .001 |  | < .001 |  |
| wolf |  | bird |  | - |  | duck |  | wolf |  | 0.004 |  | 0.006 |  | 0.727 |  | 0.467 |  | 1.000 |  |
| wolf |  | bird |  | - |  | grandpa |  | cat |  | 0.038 |  | 0.005 |  | 8.301 |  | < .001 |  | < .001 |  |
| wolf |  | bird |  | - |  | grandpa |  | duck |  | 0.028 |  | 0.005 |  | 5.675 |  | < .001 |  | < .001 |  |
| wolf |  | bird |  | - |  | grandpa |  | grandpa |  | 0.035 |  | 0.005 |  | 7.485 |  | < .001 |  | < .001 |  |
| wolf |  | bird |  | - |  | grandpa |  | wolf |  | 0.020 |  | 0.005 |  | 3.872 |  | < .001 |  | 0.032 |  |
| wolf |  | bird |  | - |  | wolf |  | cat |  | 0.026 |  | 0.005 |  | 5.369 |  | < .001 |  | < .001 |  |
| wolf |  | bird |  | - |  | wolf |  | duck |  | 0.033 |  | 0.005 |  | 6.989 |  | < .001 |  | < .001 |  |
| wolf |  | bird |  | - |  | wolf |  | grandpa |  | 0.036 |  | 0.005 |  | 7.971 |  | < .001 |  | < .001 |  |
| wolf |  | bird |  | - |  | wolf |  | wolf |  | 0.040 |  | 0.004 |  | 8.948 |  | < .001 |  | < .001 |  |
| wolf |  | cat |  | - |  | bird |  | duck |  | 0.000 |  | 0.003 |  | 0.157 |  | 0.875 |  | 1.000 |  |
| wolf |  | cat |  | - |  | bird |  | grandpa |  | -0.020 |  | 0.004 |  | -4.471 |  | < .001 |  | 0.002 |  |
| wolf |  | cat |  | - |  | bird |  | wolf |  | -0.033 |  | 0.005 |  | -6.175 |  | < .001 |  | < .001 |  |
| wolf |  | cat |  | - |  | cat |  | duck |  | 0.011 |  | 0.003 |  | 4.089 |  | < .001 |  | 0.013 |  |
| wolf |  | cat |  | - |  | cat |  | grandpa |  | 0.009 |  | 0.003 |  | 3.244 |  | 0.001 |  | 0.353 |  |
| wolf |  | cat |  | - |  | cat |  | wolf |  | -0.001 |  | 0.003 |  | -0.425 |  | 0.671 |  | 1.000 |  |
| wolf |  | cat |  | - |  | duck |  | duck |  | 0.006 |  | 0.003 |  | 2.198 |  | 0.028 |  | 1.000 |  |
| wolf |  | cat |  | - |  | duck |  | grandpa |  | 0.007 |  | 0.003 |  | 2.677 |  | 0.007 |  | 1.000 |  |
| wolf |  | cat |  | - |  | duck |  | wolf |  | -0.022 |  | 0.005 |  | -4.804 |  | < .001 |  | < .001 |  |
| wolf |  | cat |  | - |  | grandpa |  | duck |  | 0.001 |  | 0.003 |  | 0.410 |  | 0.682 |  | 1.000 |  |
| wolf |  | cat |  | - |  | grandpa |  | grandpa |  | 0.008 |  | 0.003 |  | 3.124 |  | 0.002 |  | 0.535 |  |
| wolf |  | cat |  | - |  | grandpa |  | wolf |  | -0.006 |  | 0.004 |  | -1.806 |  | 0.071 |  | 1.000 |  |
| wolf |  | cat |  | - |  | wolf |  | duck |  | 0.006 |  | 0.003 |  | 2.327 |  | 0.020 |  | 1.000 |  |
| wolf |  | cat |  | - |  | wolf |  | grandpa |  | 0.010 |  | 0.003 |  | 3.946 |  | < .001 |  | 0.024 |  |
| wolf |  | cat |  | - |  | wolf |  | wolf |  | 0.014 |  | 0.002 |  | 5.714 |  | < .001 |  | < .001 |  |
| wolf |  | duck |  | - |  | bird |  | grandpa |  | -0.026 |  | 0.004 |  | -6.267 |  | < .001 |  | < .001 |  |
| wolf |  | duck |  | - |  | bird |  | wolf |  | -0.040 |  | 0.005 |  | -7.632 |  | < .001 |  | < .001 |  |
| wolf |  | duck |  | - |  | cat |  | grandpa |  | 0.002 |  | 0.002 |  | 0.977 |  | 0.328 |  | 1.000 |  |
| wolf |  | duck |  | - |  | cat |  | wolf |  | -0.008 |  | 0.003 |  | -2.723 |  | 0.006 |  | 1.000 |  |
| wolf |  | duck |  | - |  | duck |  | grandpa |  | 0.001 |  | 0.002 |  | 0.371 |  | 0.711 |  | 1.000 |  |
| wolf |  | duck |  | - |  | duck |  | wolf |  | -0.028 |  | 0.004 |  | -6.539 |  | < .001 |  | < .001 |  |
| wolf |  | duck |  | - |  | grandpa |  | grandpa |  | 0.002 |  | 0.002 |  | 0.847 |  | 0.397 |  | 1.000 |  |
| wolf |  | duck |  | - |  | grandpa |  | wolf |  | -0.013 |  | 0.003 |  | -3.989 |  | < .001 |  | 0.020 |  |
| wolf |  | duck |  | - |  | wolf |  | grandpa |  | 0.004 |  | 0.002 |  | 1.751 |  | 0.080 |  | 1.000 |  |
| wolf |  | duck |  | - |  | wolf |  | wolf |  | 0.008 |  | 0.002 |  | 3.796 |  | < .001 |  | 0.044 |  |
| wolf |  | grandpa |  | - |  | bird |  | wolf |  | -0.044 |  | 0.005 |  | -8.503 |  | < .001 |  | < .001 |  |
| wolf |  | grandpa |  | - |  | cat |  | wolf |  | -0.012 |  | 0.003 |  | -4.300 |  | < .001 |  | 0.005 |  |
| wolf |  | grandpa |  | - |  | duck |  | wolf |  | -0.032 |  | 0.004 |  | -7.603 |  | < .001 |  | < .001 |  |
| wolf |  | grandpa |  | - |  | grandpa |  | wolf |  | -0.017 |  | 0.003 |  | -5.428 |  | < .001 |  | < .001 |  |
| wolf |  | grandpa |  | - |  | wolf |  | wolf |  | 0.004 |  | 0.002 |  | 2.165 |  | 0.030 |  | 1.000 |  |
|  | | | | | | | | | | | | | | | | | | | |

**Table 2**. Image x Music interaction. The fit scores of the correct matchings are displayed in bold.

|  | |  | |  | |  | | **95% CI** | | |  |  |
| --- | --- | --- | --- | --- | --- | --- | --- | --- | --- | --- | --- | --- |
| **Image** | | **Musical excerpt** | | **Mean** | | **SE** | | **Lower** | | **Upper** |  |  |
| **bird** |  | **bird** |  | **88.843** |  | **7.303** |  | **76.516** | **105.905** | | |  |
| cat |  | bird |  | 43.778 |  | 3.599 |  | 37.704 | 52.186 | | |  |
| duck |  | bird |  | 47.393 |  | 3.896 |  | 40.817 | 56.494 | | |  |
| grandpa |  | bird |  | 26.147 |  | 2.149 |  | 22.519 | 31.169 | | |  |
| wolf |  | bird |  | 18.846 |  | 1.552 |  | 16.227 | 22.474 | | |  |
| bird |  | cat |  | 49.509 |  | 4.084 |  | 42.619 | 59.056 | | |  |
| **cat** |  | **cat** |  | **66.161** |  | **5.448** |  | **56.968** | **78.893** | | |  |
| duck |  | cat |  | 70.747 |  | 5.815 |  | 60.931 | 84.334 | | |  |
| grandpa |  | cat |  | 65.314 |  | 5.369 |  | 56.252 | 77.857 | | |  |
| wolf |  | cat |  | 37.331 |  | 3.069 |  | 32.151 | 44.501 | | |  |
| bird |  | duck |  | 38.021 |  | 3.136 |  | 32.730 | 45.353 | | |  |
| cat |  | duck |  | 61.550 |  | 5.077 |  | 52.985 | 73.418 | | |  |
| **duck** |  | **duck** |  | **48.393** |  | **3.998** |  | **41.649** | **57.744** | | |  |
| grandpa |  | duck |  | 39.158 |  | 3.224 |  | 33.716 | 46.693 | | |  |
| wolf |  | duck |  | 49.140 |  | 4.046 |  | 42.312 | 58.597 | | |  |
| bird |  | grandpa |  | 21.499 |  | 1.767 |  | 18.515 | 25.628 | | |  |
| cat |  | grandpa |  | 55.075 |  | 4.527 |  | 47.433 | 65.652 | | |  |
| duck |  | grandpa |  | 51.313 |  | 4.232 |  | 44.172 | 61.207 | | |  |
| **grandpa** |  | **grandpa** |  | **54.239** |  | **4.458** |  | **46.713** | **64.655** | | |  |
| wolf |  | grandpa |  | 60.363 |  | 4.970 |  | 51.975 | 71.979 | | |  |
| bird |  | wolf |  | 16.591 |  | 1.364 |  | 14.288 | 19.778 | | |  |
| cat |  | wolf |  | 35.529 |  | 2.930 |  | 30.585 | 42.381 | | |  |
| duck |  | wolf |  | 20.512 |  | 1.686 |  | 17.666 | 24.452 | | |  |
| grandpa |  | wolf |  | 30.195 |  | 2.486 |  | 26.000 | 36.006 | | |  |
| **wolf** |  | **wolf** |  | **77.935** |  | **6.417** |  | **67.106** | **92.932** | | |  |

**Table 3**. The probabilities (Estimated Marginal Means from the G-MNL) of each image-musical excerpt matching. Correct couplings are displayed in bold.

|  | | | | | | | | | | | |
| --- | --- | --- | --- | --- | --- | --- | --- | --- | --- | --- | --- |
|  | | | | | | | | **95% CI** | | | |
| **Image** | | **Musical excerpt** | | **Prob.** | | **SE** | | **Lower** | | **Upper** | |
| **bird** |  | **bird** |  | **0.935** |  | **0.014** |  | **0.906** |  | **0.964** |  |
| duck |  | bird |  | 0.028 |  | 0.010 |  | 0.008 |  | 0.047 |  |
| cat |  | bird |  | 0.028 |  | 0.010 |  | 0.008 |  | 0.047 |  |
| grandpa |  | bird |  | 0.003 |  | 0.003 |  | -0.003 |  | 0.009 |  |
| wolf |  | bird |  | 0.007 |  | 0.005 |  | -0.003 |  | 0.017 |  |
| bird |  | cat |  | 0.027 |  | 0.009 |  | 0.008 |  | 0.046 |  |
| duck |  | cat |  | 0.418 |  | 0.029 |  | 0.360 |  | 0.476 |  |
| **cat** |  | **cat** |  | **0.270** |  | **0.026** |  | **0.217** |  | **0.322** |  |
| grandpa |  | cat |  | 0.252 |  | 0.025 |  | 0.200 |  | 0.303 |  |
| wolf |  | cat |  | 0.034 |  | 0.011 |  | 0.012 |  | 0.055 |  |
| bird |  | duck |  | 0.045 |  | 0.012 |  | 0.022 |  | 0.069 |  |
| **duck** |  | **duck** |  | **0.167** |  | **0.022** |  | **0.123** |  | **0.211** |  |
| cat |  | duck |  | 0.484 |  | 0.029 |  | 0.425 |  | 0.542 |  |
| grandpa |  | duck |  | 0.105 |  | 0.018 |  | 0.069 |  | 0.141 |  |
| wolf |  | duck |  | 0.199 |  | 0.023 |  | 0.152 |  | 0.246 |  |
| bird |  | grandpa |  | 0.007 |  | 0.005 |  | -0.003 |  | 0.017 |  |
| duck |  | grandpa |  | 0.293 |  | 0.027 |  | 0.239 |  | 0.347 |  |
| cat |  | grandpa |  | 0.244 |  | 0.025 |  | 0.193 |  | 0.295 |  |
| **grandpa** |  | **grandpa** |  | **0.230** |  | **0.025** |  | **0.180** |  | **0.279** |  |
| wolf |  | grandpa |  | 0.227 |  | 0.025 |  | 0.177 |  | 0.276 |  |
| bird |  | wolf |  | 0.003 |  | 0.003 |  | -0.003 |  | 0.009 |  |
| duck |  | wolf |  | 0.032 |  | 0.011 |  | 0.011 |  | 0.053 |  |
| cat |  | wolf |  | 0.041 |  | 0.012 |  | 0.018 |  | 0.065 |  |
| grandpa |  | wolf |  | 0.059 |  | 0.013 |  | 0.032 |  | 0.086 |  |
| **wolf** |  | **wolf** |  | **0.864** |  | **0.020** |  | **0.824** |  | **0.905** |  |

**Table 4**. The probabilities (Estimated Marginal Means from the G-MNL) of each word-music matching. Correct couplings are displayed in bold.

|  | | | | | | | | | | | |
| --- | --- | --- | --- | --- | --- | --- | --- | --- | --- | --- | --- |
|  | | | | | | | | **95% CI** | | | |
| **Synonyms of** | | **Musical excerpt** | | **Prob.** | | **SE** | | **Lower** | | **Upper** | |
| **bird** |  | **bird** |  | **0.918** |  | **0.016** |  | **0.886** |  | **0.951** |  |
| duck |  | bird |  | 0.044 |  | 0.012 |  | 0.020 |  | 0.068 |  |
| cat |  | bird |  | 0.034 |  | 0.011 |  | 0.013 |  | 0.055 |  |
| grandpa |  | bird |  | 0.000 |  | 0.000 |  | 0.000 |  | 0.000 |  |
| wolf |  | bird |  | 0.004 |  | 0.004 |  | -0.004 |  | 0.011 |  |
| bird |  | cat |  | 0.027 |  | 0.010 |  | 0.008 |  | 0.046 |  |
| duck |  | cat |  | 0.504 |  | 0.029 |  | 0.445 |  | 0.564 |  |
| **cat** |  | **cat** |  | **0.241** |  | **0.025** |  | **0.190** |  | **0.291** |  |
| grandpa |  | cat |  | 0.204 |  | 0.024 |  | 0.156 |  | 0.252 |  |
| wolf |  | cat |  | 0.024 |  | 0.009 |  | 0.006 |  | 0.042 |  |
| bird |  | duck |  | 0.133 |  | 0.020 |  | 0.093 |  | 0.172 |  |
| **duck** |  | **duck** |  | **0.163** |  | **0.022** |  | **0.119** |  | **0.206** |  |
| cat |  | duck |  | 0.397 |  | 0.029 |  | 0.338 |  | 0.455 |  |
| grandpa |  | duck |  | 0.101 |  | 0.018 |  | 0.065 |  | 0.136 |  |
| wolf |  | duck |  | 0.207 |  | 0.024 |  | 0.159 |  | 0.256 |  |
| bird |  | grandpa |  | 0.018 |  | 0.008 |  | 0.002 |  | 0.033 |  |
| duck |  | grandpa |  | 0.133 |  | 0.020 |  | 0.092 |  | 0.173 |  |
| cat |  | grandpa |  | 0.197 |  | 0.023 |  | 0.150 |  | 0.244 |  |
| **grandpa** |  | **grandpa** |  | **0.304** |  | **0.027** |  | **0.249** |  | **0.359** |  |
| wolf |  | grandpa |  | 0.348 |  | 0.028 |  | 0.292 |  | 0.405 |  |
| bird |  | wolf |  | 0.016 |  | 0.007 |  | 0.002 |  | 0.030 |  |
| duck |  | wolf |  | 0.024 |  | 0.009 |  | 0.006 |  | 0.042 |  |
| cat |  | wolf |  | 0.036 |  | 0.011 |  | 0.015 |  | 0.058 |  |
| grandpa |  | wolf |  | 0.055 |  | 0.013 |  | 0.028 |  | 0.082 |  |
| **wolf** |  | **wolf** |  | **0.870** |  | **0.020** |  | **0.830** |  | **0.909** |  |

**Figure 1.** Cross-cultural patterns. Fit score as a function of melody, image, and participants’ language (EN, ES, IT, ZH-S indicate English, Spanish, Italian, Chinese, respectively).


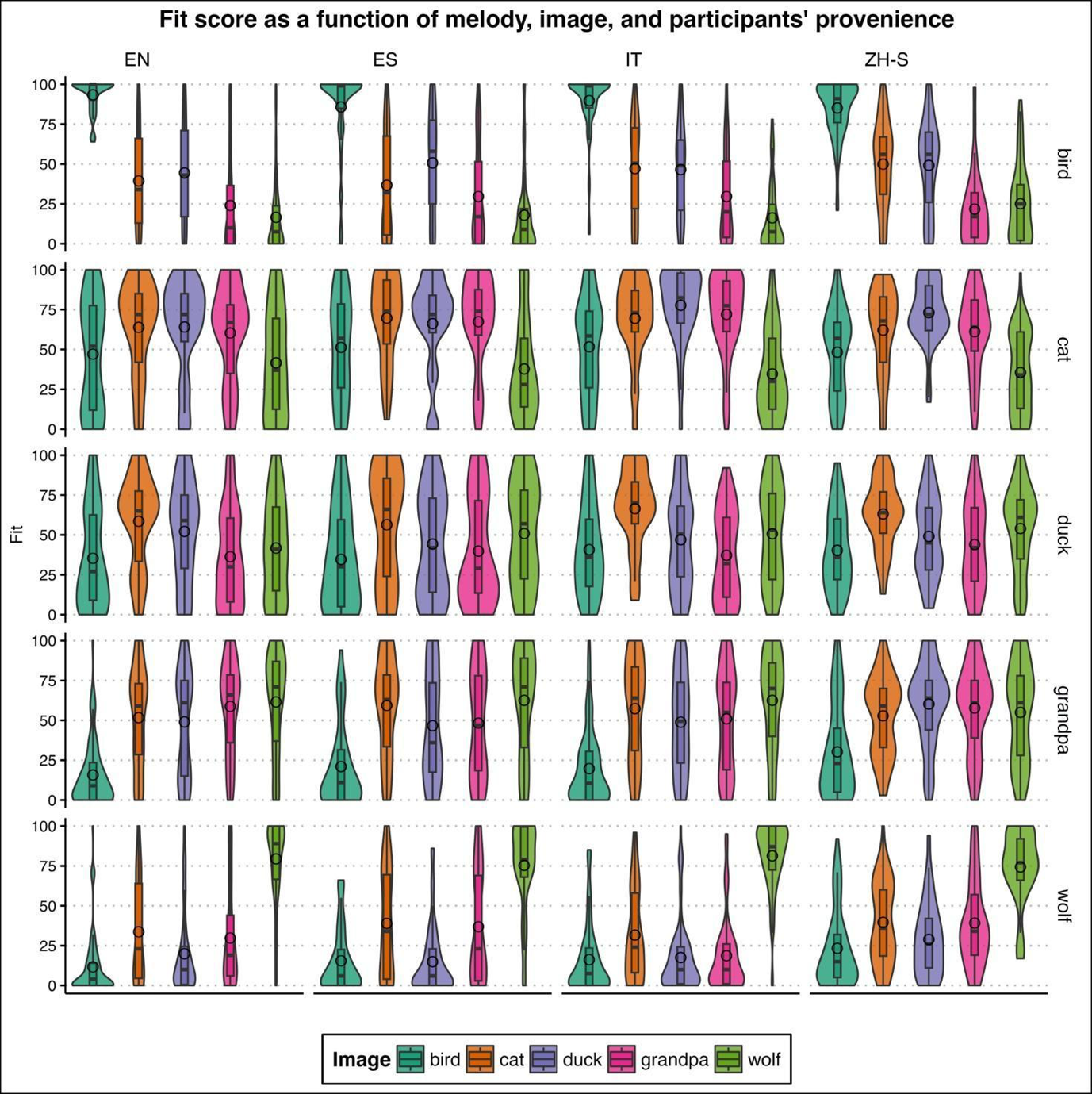


**Figure 2.** Emotional profiles of the stimuli.
